# Bioinformatic validation and machine learning-based exploration of purine metabolism-related gene signatures in the context of immunotherapeutic strategies for nonspecific orbital inflammation

**DOI:** 10.3389/fimmu.2024.1318316

**Published:** 2024-03-28

**Authors:** Zixuan Wu, Chi Fang, Yi Hu, Xin Peng, Zheyuan Zhang, Xiaolei Yao, Qinghua Peng

**Affiliations:** ^1^ Hunan University of Traditional Chinese Medicine, Changsha, Hunan, China; ^2^ Department of Ophthalmology, the First Affiliated Hospital of Hunan University of Traditional Chinese Medicine, Changsha, Hunan, China

**Keywords:** nonspecific orbital inflammation (NSOI), purine metabolism genes (PMGs), LASSO regression, SVM-RFE, bioinformatics

## Abstract

**Background:**

Nonspecific orbital inflammation (NSOI) represents a perplexing and persistent proliferative inflammatory disorder of idiopathic nature, characterized by a heterogeneous lymphoid infiltration within the orbital region. This condition, marked by the aberrant metabolic activities of its cellular constituents, starkly contrasts with the metabolic equilibrium found in healthy cells. Among the myriad pathways integral to cellular metabolism, purine metabolism emerges as a critical player, providing the building blocks for nucleic acid synthesis, such as DNA and RNA. Despite its significance, the contribution of Purine Metabolism Genes (PMGs) to the pathophysiological landscape of NSOI remains a mystery, highlighting a critical gap in our understanding of the disease’s molecular underpinnings.

**Methods:**

To bridge this knowledge gap, our study embarked on an exploratory journey to identify and validate PMGs implicated in NSOI, employing a comprehensive bioinformatics strategy. By intersecting differential gene expression analyses with a curated list of 92 known PMGs, we aimed to pinpoint those with potential roles in NSOI. Advanced methodologies, including Gene Set Enrichment Analysis (GSEA) and Gene Set Variation Analysis (GSVA), facilitated a deep dive into the biological functions and pathways associated with these PMGs. Further refinement through Lasso regression and Support Vector Machine-Recursive Feature Elimination (SVM-RFE) enabled the identification of key hub genes and the evaluation of their diagnostic prowess for NSOI. Additionally, the relationship between these hub PMGs and relevant clinical parameters was thoroughly investigated. To corroborate our findings, we analyzed expression data from datasets GSE58331 and GSE105149, focusing on the seven PMGs identified as potentially crucial to NSOI pathology.

**Results:**

Our investigation unveiled seven PMGs (ENTPD1, POLR2K, NPR2, PDE6D, PDE6H, PDE4B, and ALLC) as intimately connected to NSOI. Functional analyses shed light on their involvement in processes such as peroxisome targeting sequence binding, seminiferous tubule development, and ciliary transition zone organization. Importantly, the diagnostic capabilities of these PMGs demonstrated promising efficacy in distinguishing NSOI from non-affected states.

**Conclusions:**

Through rigorous bioinformatics analyses, this study unveils seven PMGs as novel biomarker candidates for NSOI, elucidating their potential roles in the disease’s pathogenesis. These discoveries not only enhance our understanding of NSOI at the molecular level but also pave the way for innovative approaches to monitor and study its progression, offering a beacon of hope for individuals afflicted by this enigmatic condition.

## 1 Introduction

Nonspecific orbital inflammation (NSOI) emerges as a benign, non-infectious orbital disorder, devoid of any discernible systemic or local causation. Accounting for 6%-16% of all ocular pathologies and 11% of orbital malignancies, NSOI predominantly afflicts middle-aged individuals, with a notable predisposition among women (1, 2). The pathophysiological roots of NSOI remain shrouded in uncertainty. However, recent investigations hint at its potential linkage with a constellation of factors, including Streptococcal pharyngitis, viral upper respiratory infections, and a gamut of autoimmune phenomena such as rheumatologic conditions, multifocal fibrosis, and Crohn’s disease (2, 3). Manifestations of NSOI are heterogenous, ranging from lacrimal gland inflammation and extraocular muscle myositis to other less common presentations (4). Systemic corticosteroids stand as the cornerstone of NSOI management, often employed as a first-line intervention upon a presumptive diagnosis. Yet, the chronic use of corticosteroids is marred by an array of adverse effects (5). Despite the initial effectiveness of corticosteroid therapy, the recurrence rate of NSOI alarmingly exceeds 50%. This underscores the urgent need for an enhanced understanding of NSOI’s molecular landscape, aiming to develop innovative therapeutic modalities that curtail recurrence and improve patient outcomes (6). In the realm of biological systems, the assimilation of nutrients and execution of metabolic functions are fundamental for survival. A defining characteristic of cancer is its ability to reprogram metabolism, thereby supporting tumor cell proliferation and survival. Recent studies reveal that oncogenic transformation fosters a distinct metabolic phenotype in cancer cells, which, in turn, remodels the immune microenvironment. This complex environment, characterized by a diverse array of cell types, often suffers from inadequate oxygen and nutrient availability, a consequence of poorly developed or aberrantly differentiated vasculature (7). As scientific exploration progresses, the study of non-tumoral immune infiltrates gains prominence, propelled by accumulating evidence that links immune responses to significant metabolic shifts within tissues. These shifts encompass nutrient depletion, increased oxygen consumption, and the production of reactive nitrogen and oxygen species (8). Concurrently, the microenvironmental milieu profoundly influences immune cell differentiation and function, suggesting that metabolic interventions could enhance the effectiveness of immunotherapeutic strategies (9).

In the vast and intricate web of biological existence, the assimilation of nutrients and the execution of metabolic processes stand as foundational pillars. Among these metabolic derivatives, purines distinguish themselves as quintessential to the very essence of life, providing the elemental substrates for the synthesis of DNA and RNA (10). Yet, their significance extends far beyond the genomic sphere, as purines are integral to the composition of critical biomolecules such as ATP, GTP, cAMP, NADH, and coenzyme A. These molecules play instrumental roles in a variety of cellular functions, including energy production, signal transduction, redox metabolism, and the biosynthesis of fatty acids (11). Furthermore, purines are vital in the regulation of immune responses and in facilitating the intricate interactions between host and pathogen. Mammalian cells orchestrate purine metabolism via two primary pathways: *de novo* synthesis and the salvage pathway, the latter of which predominates in meeting the cellular demands for purines by recycling degraded bases. In the context of rapidly proliferating tumor cells, the requirement for purines intensifies, leading to an upregulation of the *de novo* synthesis pathway (12). This heightened demand for purines in tumor cell replication has positioned purine antimetabolites at the forefront of anticancer therapeutics, marking a seminal advancement in the treatment of malignancies such as acute lymphocytic leukemia, acute myeloid leukemia, and chronic myeloid leukemia (13). This paradigm shift towards leveraging purine antimetabolites in the realm of nonneoplastic diseases underscores their potential in modulating disease progression by inhibiting DNA synthesis and curtailing cell growth. The recent discovery of purinosomes, entities intimately linked with the cell cycle and purine metabolism, heralds a novel therapeutic vista that emphasizes targeting purine metabolic pathways and purinosome formation (14). While integrating purine metabolic strategies with immunotherapy emerges as a promising avenue for managing NSOI, the landscape of purine metabolism within the context of immunogenicity and immunotherapeutic interventions remains largely unexplored. Driven by this conspicuous knowledge void, our investigation endeavors to provide a comprehensive evaluation of PMGs within the ambit of immunotherapy for NSOI. By delving into this uncharted territory, we aim to illuminate new pathways and therapeutic targets, potentially revolutionizing the approach to NSOI management and offering a beacon of hope for patients afflicted by this condition.

**Figure 1:**
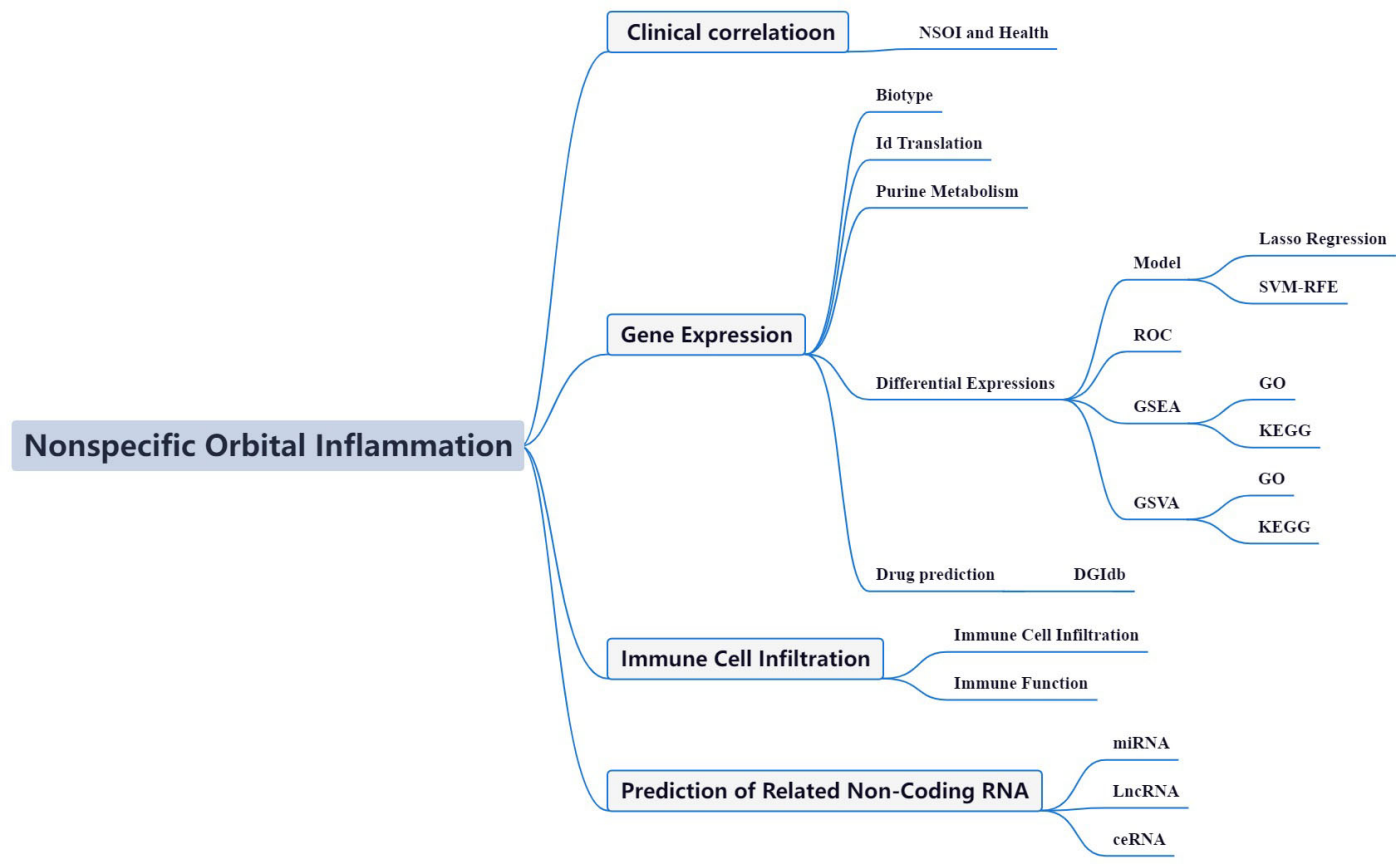
Framework. In pursuit of a deeper comprehension of NSOI, we embarked on an investigative journey by harnessing patient-derived datasets curated from the GEO repositories. Specifically, we leveraged the GSE58331 dataset as our principal cohort, supplemented by the GSE105149 dataset for validation purposes. Employing a meticulous matching strategy of PMGs, we embarked on differential expression analyses and subsequent construction of a prognostic risk model. Our methodological framework unearthed a distinct subset of PMGs exhibiting prognostic relevance in the landscape of NSOI, thereby underscoring their candidacy as promising biomarkers. To unravel the functional underpinnings of these identified genes, we executed an exhaustive suite of bioinformatics analyses spanning GO, KEGG, and GSEA. These comprehensive analyses were augmented by seamless integration with diverse databases, furnishing a multi-dimensional portrayal of the implicated PMGs within the intricate milieu of cellular processes, signaling cascades, and gene regulatory networks. Furthermore, in our quest to illuminate the immunological landscape and glean insights into transcriptional dynamics, we conducted an intricate evaluation encompassing immune cell infiltration patterns, functional alterations, and transcriptomic variations. This holistic approach not only enriches our comprehension of the implications of PMGs in NSOI but also lays a robust foundation for the delineation of prospective targeted therapeutic interventions in the management of this enigmatic neurological disorder.

The advent of high-throughput data analysis, viewed through the lens of bioinformatics, has become a cornerstone in unraveling the complex gene networks underlying diverse disease states, offering invaluable insights for molecular and mechanistic investigations (15). Within this scientific milieu, the NSOI Initiative’s extensive collection of high-throughput transcriptomic sequencing data, enriched with comprehensive clinical annotations, presents a unique vantage point for examining the transcriptional deviations and associated molecular pathways integral to NSOI (16). Such bioinformatic inquiries have begun to illuminate the multifaceted pathophysiology of NSOI, providing a richer understanding of the condition from various angles. Despite these advances, a significant gap in our knowledge remains—the involvement of PMGs in NSOI has yet to be explored using bioinformatic methodologies (17, 18). It is against this backdrop that our study aims to traverse the NSOI landscape within the GEO, specifically focusing on the role of PMGs, a journey visually encapsulated in **Figure 1**. This endeavor seeks to deepen our comprehension of NSOI, potentially uncovering novel molecular targets for therapeutic intervention and advancing our grasp of its underlying biological mechanisms.

## 2 Materials and methods

We adopted the methodologies delineated by Zi-Xuan Wu and colleagues in 2023 (19).

### 2.1 Raw data acquisition

The foundational datasets for our investigation were sourced from the GEO series: GSE58331 and GSE105149, utilizing the GPL570-55999 platform for consistency. The GSE58331 dataset was designated for training purposes, while GSE105149 was reserved for validation, ensuring a robust framework for our analysis. Additionally, the MSigDB was consulted to compile a comprehensive list of 175 PMGs, as detailed in **Supplementary Table S1**.

### 2.2 Delineation of Differentially Expressed Genes

Our methodology for extracting precise mRNA profiles involved the utilization of Perl scripts to meticulously match and sort transcriptional data from the GSE58331 dataset. Following normalization procedures, we applied stringent criteria for identifying differential expression among PMGs: FDR<0.05 and |log2FC|≥1. This rigorous approach enabled the isolation of significantly altered PMGs for further scrutiny. To elucidate the intricate relationships among these genes, Pearson’s correlation coefficient was harnessed, leveraging the corrplot package in R for comprehensive correlation analysis. This step was pivotal in highlighting genes with statistically significant associations within the identified modules.

### 2.3 Functional enrichment analysis: GO and KEGG pathways

To unravel the biological implications and pathway involvements of the DEGs, we embarked on Gene Ontology (GO) and Kyoto Encyclopedia of Genes and Genomes (KEGG) analyses. Utilizing R, we delved into the impact of differentially expressed PMGs on biological processes (BP), molecular functions (MF), and cellular components (CC). This exploration was aimed at delineating the overarching biological themes and molecular pathways that these genes influence, providing a deeper understanding of their roles in the context of disease pathology and offering insights into potential therapeutic targets. Through this multi-faceted analysis, we sought not only to categorize the DEGs but also to illuminate the complex interplay between purine metabolism and its broader biological and clinical significance.

### 24 Building a model and immune cell infiltration

In our quest to develop a predictive model of unmatched precision and dependability, we employed the glmnet package for Lasso regression analysis, augmented by cross-validation, to refine our model. This approach effectively reduced overfitting and improved the model’s predictive capability for complex biological datasets. To further validate our model, we utilized the sophisticated SVM-RFE algorithm with the e1071 package, meticulously constructing a machine learning model. Cross-validation played a critical role in evaluating the model’s error rates and accuracy, ensuring its robustness and reliability. The Random Forest algorithm, celebrated for its ensemble learning efficacy, was integral to our analysis. By generating multiple decision trees and amalgamating their predictions, it minimized overfitting risks and enhanced model generalization. This method’s distinctive feature—random feature selection and bootstrap sampling—promoted diversity among decision trees, improving the model’s overall accuracy. Through the use of the randomForest and ggplot2 packages, we focused on analyzing differentially expressed genes, identifying key genes essential for disease classification. In the concluding phase, we ranked the significance of these feature genes using an integrated approach that combined insights from Lasso regression, Random Forest, and SVM models, providing a nuanced understanding of their roles in disease pathology. Moreover, the CIBERSORT algorithm allowed us to analyze the immune cell composition, offering deeper insights into the immune landscape pertinent to the disease. This comprehensive and rigorous analytical approach not only augmented the accuracy of disease classification but also opened new avenues for understanding the disease’s molecular basis.

### 2.5 Gene set enrichment and variation analyses

To discern the functional dynamics and pathway alterations across a spectrum of samples, Gene Set Enrichment Analysis (GSEA) and Gene Set Variation Analysis (GSVA) were deployed. These analyses facilitated the identification of functionally related gene sets and pathway changes, utilizing scores and visual representations to elucidate the active biological processes and pathways within different risk stratifications. Employing R, we delved into the impact of differentially expressed PMGs on BP, MF, CC, and pathways, offering a granular understanding of their roles in disease mechanisms.

### 2.6 Drug-gene interaction insights

With the maturation of bioinformatics, the construction of biological models and the identification of effective biomarkers have become pivotal in diagnosing diseases. However, the transition from biomarker identification to clinical application remains a critical challenge. Thus, the prediction of therapeutic interventions based on these markers is anticipated to play a vital role in the future management and treatment of NSOI. Recognizing validated biomarkers as cornerstones for therapeutic strategies, precise drug prediction becomes paramount. To this end, we utilized the DGIdb to forecast potential drug interactions for the hub genes identified, laying the groundwork for targeted therapeutic interventions.

### 2.7 Interplay between common miRNAs and lncRNAs

The regulatory landscape of genetics is significantly influenced by non-coding RNA transcripts, including microRNAs (miRNAs) and long non-coding RNAs (lncRNAs). miRNAs can modulate gene expression through the enhancement or repression of mRNA degradation and translation, while lncRNAs, comprising over 200 nucleotides, orchestrate a plethora of cellular processes through mechanisms like chromosomal modifications, transcriptional activation, and interference. Recent studies highlight the extensive interplay between miRNAs and lncRNAs, fostering a competitive binding scenario among miRNAs, lncRNAs, and other regulatory entities. This interaction has unveiled the concept of competitive endogenous RNAs (ceRNAs), where an lncRNA may regulate gene expression by sequestering miRNAs. In light of these findings, our investigation seeks to determine whether specific miRNAs and lncRNAs share regulatory mechanisms and developmental pathways in NSOI, potentially unveiling novel avenues for understanding and treating this complex condition.

### 2.8 Construction of an mRNA-miRNA-lncRNA network

To delineate the interactive landscape among mRNA, miRNA, and lncRNA entities within NSOI, we sourced target gene information from miRTarBase and PrognoScan, databases providing empirically validated miRNA-lncRNA-target interactions. The regulated network, established by intersecting the target genes of common mRNA-miRNA-lncRNA with NSOI-associated genes, was visualized using Cytoscape software, offering a graphical representation of the molecular interplay critical for disease pathophysiology.

### 2.9 Mendelian randomization analysis

To ensure the independence of exposure and outcome variables in our genome-wide association study (GWAS) summary data, we engaged in an association analysis via the TwoSampleMR package in R. Designating TNF-related expression as the exposure and ovarian function diminution as the outcome, we aimed to explore potential causal relationships. The analysis entailed: 1. Instrumental Variables (IVs) Configuration: TNF-related expressions were screened with a P-value threshold of < 5×10^-8 to identify strongly associated exposures. 2. Independence Configuration: Linkage disequilibrium (LD) between SNPs was calculated using the PLINK clustering method, excluding SNPs with LD coefficient r^2 > 0.001 and within 10,000 kb to ensure SNP independence and reduce pleiotropic biases. 3. Statistical Strength Configuration: The robustness of instrumental variables was assessed using the F-statistic (F = β^2/SE^2), with variables having F < 10 deemed inadequate to mitigate confounding effects.

Leveraging GWAS data, SNPs associated with the instrumental variables were identified, and through the “harmonise_data” function within TwoSampleMR, we aligned allelic directions of exposure and outcome, excluding incompatible SNPs. The inverse variance-weighted (IVW) method served as the cornerstone for causal inference, employing the variance of instrumental variables as weights to determine causal dynamics, thereby advancing our understanding of the genetic architecture underlying disease states.

## 3 Results

### 3.1 DEG identification and principal component analysis

**Figure 2:**
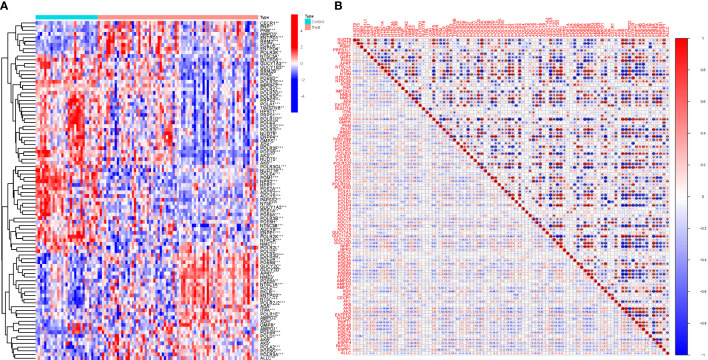
Principal component analysis. **(A)** Analysis of difference. **(B)** Analysis of correlation.

Among the 92 PMGs, some PMGs were found to be significantly different. In addition, Some genes cluster in the treat group and some in the control group. Treat: NME7, POLR2L, POLD2, POLR3D, POLD4, PDE6B, GUCY2C, etc. Control: ENTPD5, GUCY1A3, GUCY1B3, RRM2B, PFAS, PDE6D, etc. (**Figure 2A**). These PMGs were tested for correlation and a visualization of the correlation matrix was constructed (**Figure 2B**; **Supplementary Table S2**).

### 3.2 Enrichment analysis of PMGs

**Figure 3:**
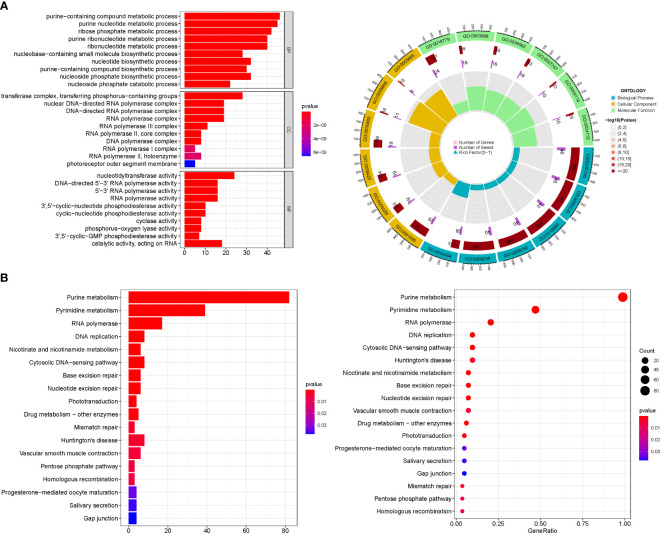
For PMGs, GO, and KEGG analyses were performed. **(A)** The GO circle illustrates the scatter map of the selected gene's logFC. **(B)** The KEGG barplot and bubble illustrates the scatter map of the logFC of the indicated gene.

GO enrichment analysis revealed 436 core targets, including BP, MF, and CC. The MF mainly involves guanyl nucleotide binding (GO:0019001), guanyl ribonucleotide binding (GO:0032561). The CC mainly involves cell projection membrane (GO:0031253), vesicle lumen (GO:0031983). The BP mainly involves neutrophil activation (GO:0042119), neutrophil mediated immunity (GO:0002446). KEGG enrichment analysis revealing that the over-expressed genes were mainly involved in Huntington’s disease (hsa05016), Purine metabolism (hsa00230), Vascular smooth muscle contraction (hsa04270) (**Figure 3**; **Supplementary Tables S3A, B**).

### 3.3 Construction of the model

**Figure 4:**
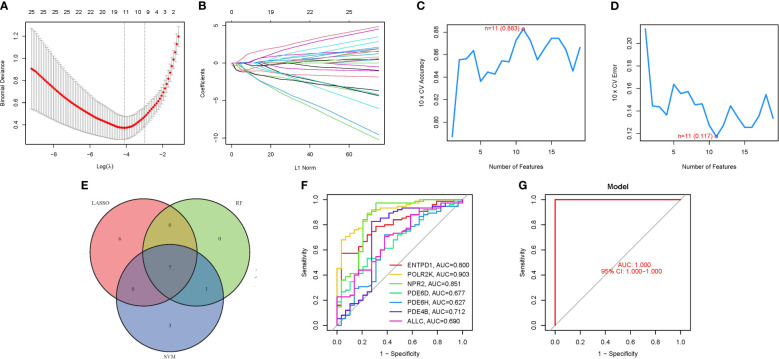
The development of the PMGs signature. **(A)** Regression of the 7 NSOI-related genes using LASSO. **(B)** Cross-validation is used in the LASSO regression to fine-tune parameter selection. **(C, D)** Accuracy and error of this model. **(E)** Venn. **(F)** AUC of 7 hub genes. **(G)**: AUC of train group.

To delineate a gene signature with pronounced predictive utility, we employed a methodological trifecta comprising LASSO regression analysis, Cox proportional hazards regression analysis, and the identification of an optimal cutoff value, as depicted in **Figures 4A, B**. The construction of a machine learning model via SVM-RFE ensued, serving to validate the model’s predictive accuracy and reliability. This model demonstrated a commendable accuracy of 0.883, alongside a minimal error rate of 0.117 (**Figures 4C, D**). A cross-reference of seven PMGs identified through both LASSO and SVM methodologies is presented in **Figure 4E**. Subsequent comparative analysis with the 7 hub genes revealed significantly high AUC values, indicating robust predictive power: ENTPD1 (AUC=0.800), POLR2K (AUC=0.903), NPR2 (AUC=0.851), PDE6D (AUC=0.677), PDE6H (AUC=0.627), PDE4B (AUC=0.712), and ALLC (AUC=0.690) (**Figure 4F**). Notably, an AUC of 1.000 (95% Confidence Interval: 1.000−1.000) was achieved in the GSE58331 dataset, underscoring the model’s exceptional precision and stability (**Figure 4G**; **Supplementary Table S4**).

In addressing concerns regarding the AUC value, it is imperative to highlight the observed AUC of 0.883, as evidenced in **Figure 4**. This metric unequivocally attests to the high accuracy of our predictive model. While individual genetic variability may contribute to fluctuations in AUC values, it is crucial to emphasize that the aggregated AUC values for the evaluated genes consistently approximate or exceed the notable threshold of 0.7. This synthesis of results substantially bolsters the validity, precision, and resilience of our predictive framework, affirming its potential utility in clinical and research settings (**Table 1**).

**Table 1:** The characteristics of model.

Label	LASSO	SVM-RFE	Random Forest
Sensitivity	1	1	0.818181818
Specificity	0.965517241	1	0.785714286
Pos Pred Value	0.916666667	1	0.75
Neg Pred Value	1	1	0.846153846
Precision	0.916666667	1	0.75
Recall	1	1	0.818181818
F1	0.956521739	1	0.782608696
Prevalence	0.275	0.275	0.44
Detection Rate	0.275	0.275	0.36
Detection Prevalence	0.3	0.275	0.48
Balanced Accuracy	0.982758621	1	0.801948052

### 3.4 GSEA of analysis

**Figure 5:**
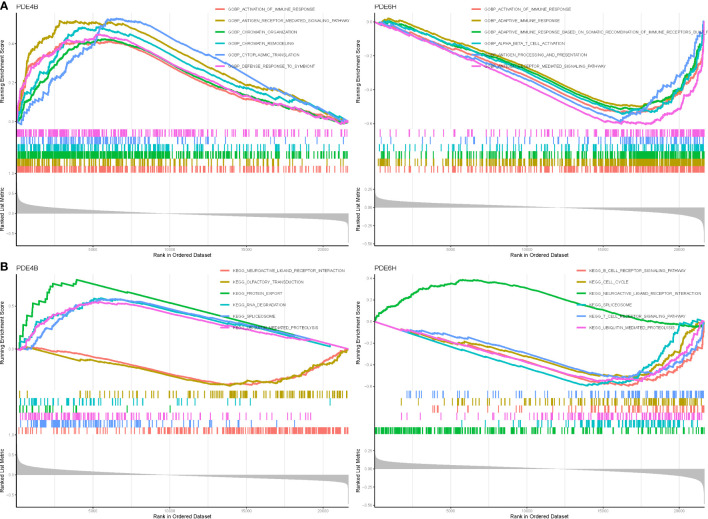
GSEA of Analysis in PDE4B and PDE6D. **(A)** GO. **(B)** KEGG.

We discovered that PDE4B and PDE6H may be the most related genes with NSOI by evaluating the literature and the sensitivity of these hub genes in the model. In terms of BP in GO analysis, PDE4B mainly involves antigen receptor mediated signaling pathway, chromatin remodeling, activation of immune response. PDE6H mainly involves adaptive immune response, alpha beta t cell activation, gobp adaptive immune response based on somatic recombination of immune receptors (**Figure 5A**). In terms of kegg analysis, PDE4B mainly involves protein export, rna degradation, ubiquitin mediated proteolysis. PDE6H mainly involves t cell receptor signaling pathway, ubiquitin mediated proteolysis, rna degradation (**Figure 5B**; **Supplementary Table S5**).

### 3.5 Immune cells

**Figure 6:**
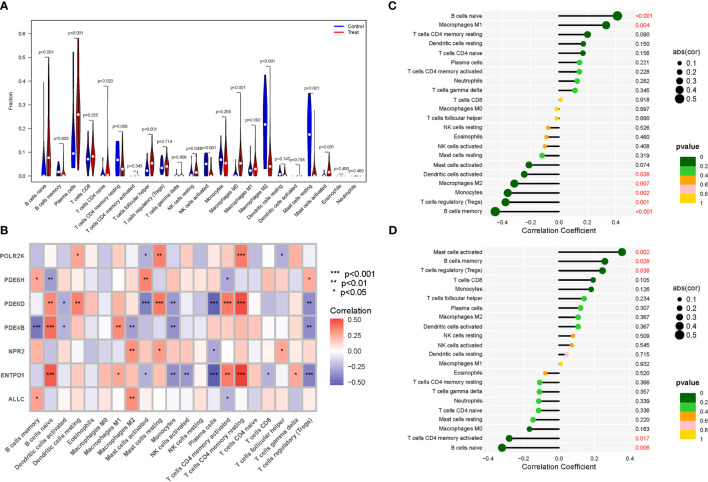
Expression of Immune cells. **(A)** Expression of immune cells in different clusters. **(B)** Correlation between PMGs and immune cells. **(C)** PDE4B. **(D)** PDE6H.

The immunological environment has a critical role in the initiation and progression of NSOI. We created a vioplot to display the outcomes of immune cells. B cells naive, B cells memory, T cells follicular helper, NK cells resting, Macrophages M0, and Mast cells activated were highly expressed in the treat group. The control group had considerably larger levels of cells memory, NK cells activated, Macrophages M2, and Mast cells resting (**Figure 6A**). We also performed a correlation study of these genes and immune cells (**Figure 6B**). In addition, we analyzed the immune infiltration of PDE4B and PDE6H separately (**Figures 6C, D**).

### 3.6 GSVA of analysis

**Figure 7:**
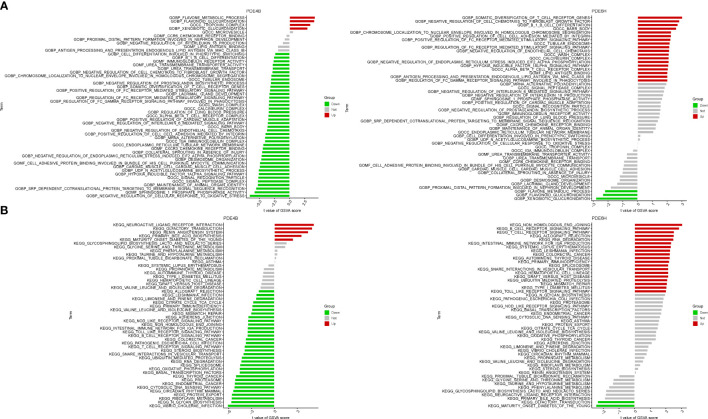
GSVA of Analysis in PDE4B and PDE6D. **(A)** GO. **(B)** KEGG.

In terms of GO analysis, PDE4B mainly involves BP flavone metabolic process, BP flavonoid glucuronidation, and CC troponin complex. PDE6D mainly involves BP somatic diversification of t cell receptor genes, BP negative regulation of cell chemotaxis to fibroblast growth factor, CC barr body, CC tubular endosome, and MF lipid antigen binding (**Figure 7A**). In terms of KEGG analysis, PDE4B mainly involves neuroactive ligand receptor interaction, olfactory transduction, renin angiotensin system. PDE6H mainly involves non homologous end joining, B and T cell receptor signaling pathway (**Figure 7B**).

### 3.7 Drug-gene interactions

**Figure 8:**
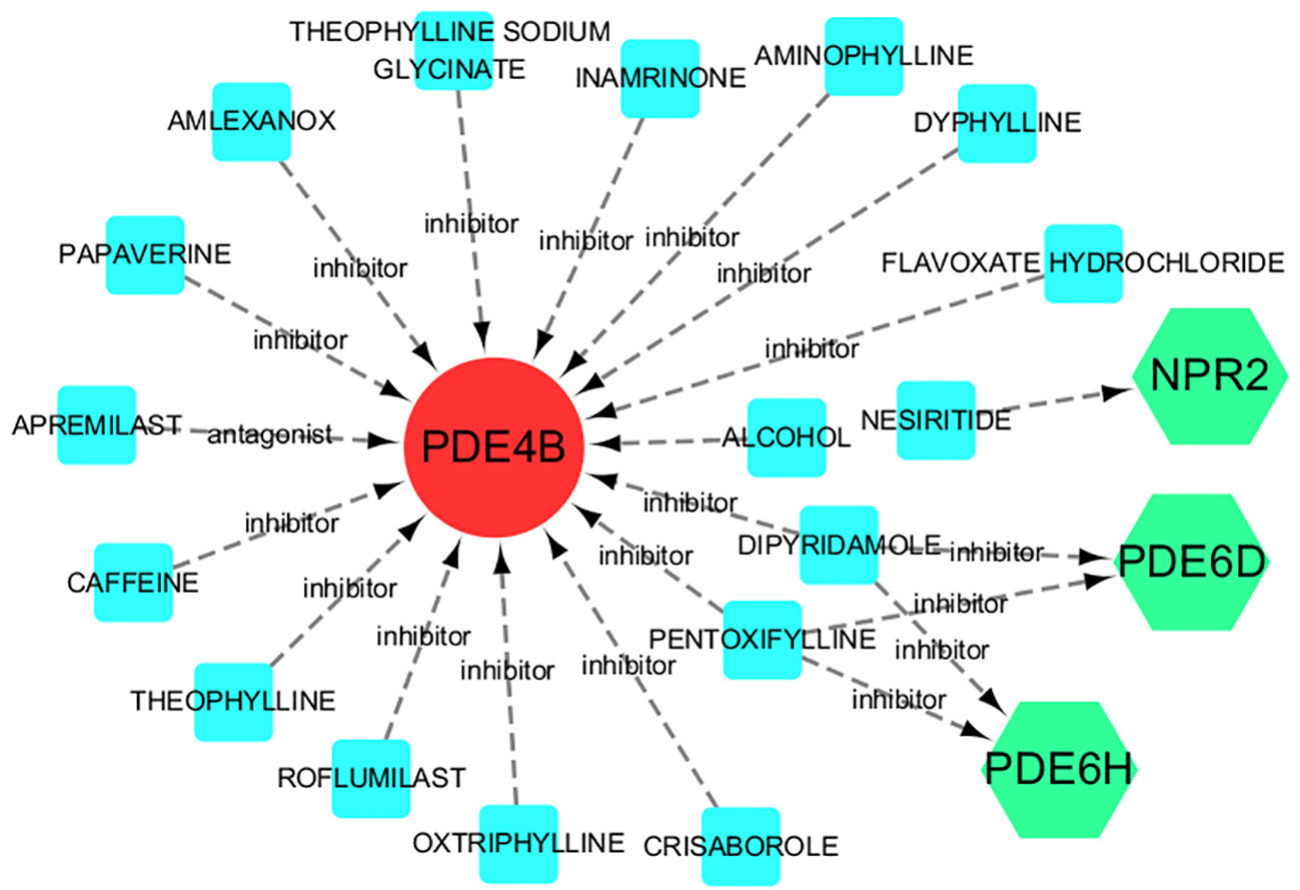
Drug-gene interactions. Red circles are up-regulated genes, green hexagons are down-regulated genes, and blue squares are associated drugs.

The seven hub genel predicted 17 drugs. These include NESIRITIDE, DIPYRIDAMOLE, PENTOXIFYLLINE, AMINOPHYLLINE, ROFLUMILAST, etc. (**Supplementary Table S6**). In addition, the drug-gene interactions were visualized by Cytoscape 3.7.1 (**Figure 8**).

### 3.8 Identification of common RNAs and construction of miRNAs-lncRNAs shared genes network

**Figure 9:**
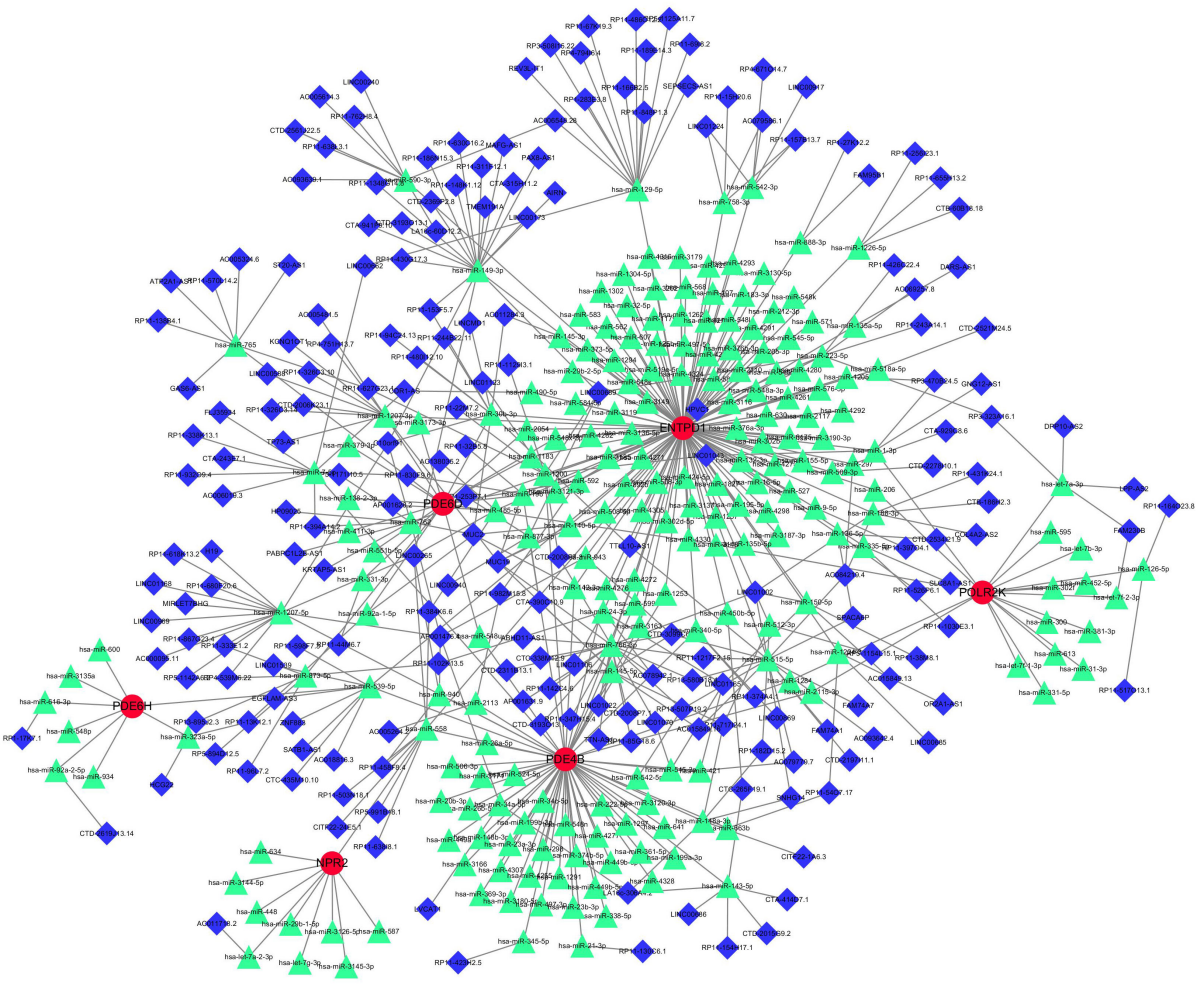
miRNAs-LncRNAs shared Genes Network. Red circles are mrnas, blue quadrangles are miRNAs, and green triangles are lncRNAs.

Three databases were searched for 242 miRNAs and 284 lncRNAs linked with NSOI (**Supplementary Tables S7A** **, B**). **Supplementary Table S7** shows the matching of these genes against the corresponding miRNA database. These databases include miRanda (20), miRDB (21), and TargetScan (22). When the corresponding database matched the relevant miRNA, the score was marked as 1. It can be seen that when all three databases can be matched, it is 3 points. The miRNA was matched by spongeScan database (23) to obtain the corresponding lncRNA data. The network of miRNAs-lncRNAs-genes was constructed by taking the intersection of them and shared genes (obtained by Lasso regression, Random forest and SVM-RFE). Finally, the miRNAs-genes network included 207 lncRNAs, 216 miRNAs, and several common genes, including six hub genes (ENTPD1, PDE4B, PDE6H, PDE6D, POLR2K, and NPR2) (**Figure 9**).

### 3.9 Validation of hub genes

**Figure 10:**
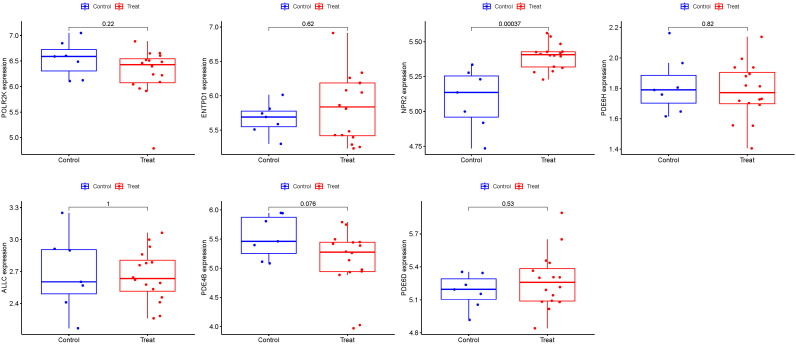
Seven hub genes were validated.

GSE105149 was used for validation to boost our model’s confidence and prediction accuracy of these hub genes. However, However, among these Seven PMGs, only NPR2 showed significant differences in GSE105149 analysis (**Figure 10**). When we recalibrated the data, we found that the sample sizes in the two data sets differed, as did the sources of patients. This may have contributed to the bias in the results.

### 3.10 Model verification

**Figure 11:**
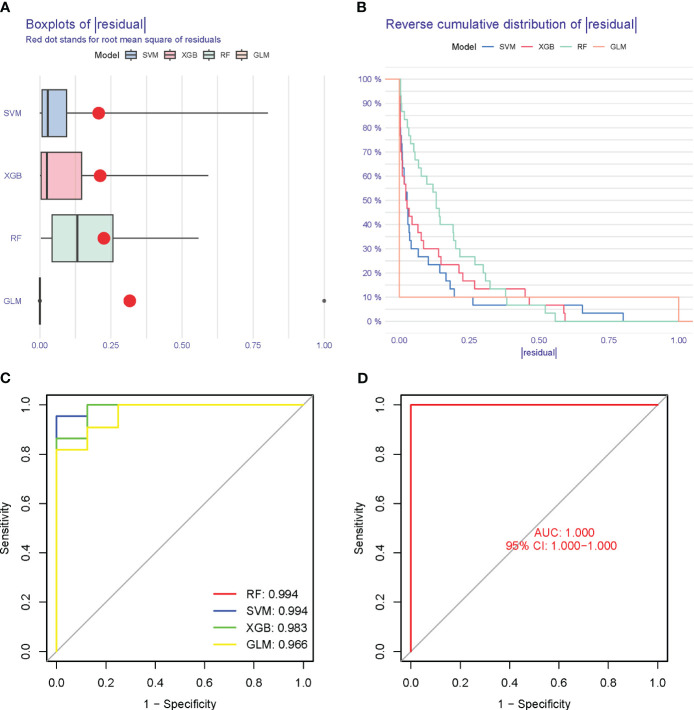
Model verification. **(A)** Residual expression patterns. **(B)** Model expression patterns **(C)** AUC of model. **(D)** AUC of test group.

The Boxplots depicted the residual expression patterns of these genes in NSOI (**Figure 11A**). There are some differences in the proportions of the four different modes (**Figure 11B**). The GlnMgs’ diagnostic capacity in distinguishing NSOI from control samples revealed a satisfactory diagnostic value, with an AUC of RF: 0.994; SVM: 0.994; XGB: 0.983; GLM: 0.966 (**Figure 11C**). An AUC of 1.000 (95% CI 1.000-1.000) in GSE105149 (**Figure 11D**).

### 3.11 Mendelian randomization analysis

**Figure 12:**
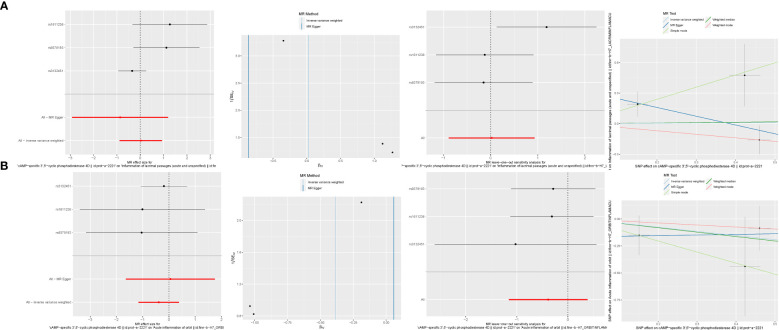
Mendelian randomization analysis. **(A)** PDE4B. **(B)** PDE6H.

In our exploration of the intrinsic connection between PDE4B, PDE6H, and NSOI, forest plots were meticulously employed to visually articulate the associations. For PDE4B, the SNP rs3132451 conspicuously positioned itself to the right of the confidence interval, indicating a positive association. Conversely, rs1611236 and rs9378193 were observed to the left, reinforcing the credibility of our findings (**Figure 12A**). In the case of PDE6H, SNPs rs9378193, rs1611236, and rs3132451 were all situated to the right of the confidence interval (**Figure 12B**), suggesting a similar trend of association with NSOI. Further dissecting the heterogeneity inherent in our analysis, the funnel plot tailored to NSOI revealed a deviation from the expected symmetrical distribution, albeit maintaining a general symmetry. This nuanced observation was further scrutinized through sensitivity analysis, employing a “leave-one-out” approach. Remarkably, the omission of any individual SNP from the analysis had a negligible effect on the results of the Inverse Variance Weighted (IVW) analysis, indicating that the remaining SNPs consistently mirrored the outcomes of the aggregate dataset. Substantiating the validity of our findings, the MR-Egger regression analysis was invoked, providing a solid foundation that bolsters both the robustness and authenticity of our results and the methodologies applied. This Mendelian randomization analysis unequivocally confirms the intimate association of PDE4B and PDE6H with NSOI. Hence, it delineates a potential pathway to modulate the incidence, evolution, and progression of NSOI by intervening in the functions of PDE4B and PDE6H, presenting a promising avenue for therapeutic intervention and a deeper understanding of the disease mechanism.

## 4 Discussions

NSOI represents a rare, idiopathic ocular anomaly characterized by unilateral, severe orbital edema, emerging independently of viral or systemic conditions, with the potential for aggravation through optic nerve impairment. Despite its distinct clinical presentation, the precise molecular underpinnings of NSOI remain largely elusive (24). Central to unraveling this conundrum is the regulation of gene expression, which is anticipated to play a pivotal role in the pathophysiology of NSOI. Purines, encompassing a class of heterocyclic bicyclic aromatic compounds, are integral to numerous metabolic and cell signaling processes. The enzymatic machinery governing the anabolic and catabolic pathways of purine nucleotides has been implicated in the proliferation of tumor cells and their resistance to therapies (25). A dysregulation between the antioxidant and pro-inflammatory roles of uric acid may serve as a catalyst for tumor initiation and progression. Moreover, disturbances in purine nucleotide metabolism, through their influence on signal transduction pathways, are capable of inducing changes in gene and protein expression profiles. These alterations can enhance cellular malignancy, invasiveness, and the tendency to metastasize (26). Recent research has identified several risk markers across various ocular diseases, yet, without thorough validation and extensive replication, these findings remain largely theoretical in their application (27). Historically, the focus of research has predominantly been on the impact of singular purine metabolism regulators within the context of cancer. However, the collective influence of multiple genes associated with purine metabolism in non-cancerous diseases has not been adequately explored (28). With the evolving understanding of tumor biology, there is a noticeable shift towards investigating the non-tumoral aspects of diseases. In the case of NSOI, examining the complex patterns of purine metabolism during disease progression could shed light on the critical role of purine metabolism within NSOI’s pathogenesis. Such insights hold the promise of informing targeted therapeutic strategies, thereby opening new avenues for the management and treatment of NSOI.

In NSOI, we discovered 92 DEGs linked to Purine Metabolism. We discovered the PMGs by intersecting DEGs, Lasso regression and SVM-RFE after learning more about the involvement of PMGs in NSOI. Through crossover analysis, we discerned seven pivotal PMGs (ENTPD1, POLR2K, NPR2, PDE6D, PDE6H, PDE4B, and ALLC). These hub genes emerged as having substantial diagnostic potential for NSOI, a conclusion reinforced by validation using external datasets, thus underlining their critical role in the disease’s pathogenesis. Notwithstanding these insights, our research also revealed a significant knowledge gap regarding the interactions of these genes with specific transcription factors within the PMG framework. During our extensive literature review, particular attention was drawn to PDE4B and PDE6H, which appeared to be pivotal in the interplay between NSOI and PMGs. Further exploration of their biological roles illuminated their involvement in a spectrum of immune-related processes, including peroxisome targeting sequence binding, seminiferous tubule development, and ciliary transition zone. This insight suggests that PMGs might exert extensive regulatory effects over a variety of biological pathways, with a notable emphasis on immune-related pathways. Such a regulatory impact could profoundly influence the pathophysiological development of NSOI. Our findings imply that these genes may be fundamental in deciphering the progression of NSOI and could unveil new pathways for therapeutic intervention, offering promising prospects for future research and treatment modalities in this field.

In our investigation into NSOI, we identified 92 DEGs intimately associated with purine metabolism. This discovery emerged from an integrative analysis combining DEGs with advanced computational techniques, including Lasso regression and SVM-RFE, to underscore the significance of PMGs in NSOI’s pathology. Through meticulous crossover analysis, we pinpointed seven central PMGs (ENTPD1, POLR2K, NPR2, PDE6D, PDE6H, PDE4B, and ALLC) as harboring significant diagnostic potential for NSOI. This assertion gains further credence through validation against external datasets, thereby affirming their fundamental role in the disease’s molecular landscape. Despite these advances, our exploration laid bare a notable void in our understanding of how these genes interact with specific transcription factors within the purine metabolism milieu. A focused review of existing literature brought to light the genes PDE4B and PDE6H as particularly critical in the NSOI-PMG nexus. Delving deeper into their biological functions revealed their engagement in diverse immune-related processes, spanning from peroxisome targeting sequence binding and seminiferous tubule development to the ciliary transition zone organization. These findings intimate that PMGs could wield broad regulatory sway across myriad biological pathways, with a pronounced focus on immune modulation. The implications of our research are twofold. First, it posits these genes as pivotal in unraveling the complex pathophysiological tapestry of NSOI, thereby presenting them as key targets for further scientific inquiry. Second, it opens new horizons for therapeutic exploration, suggesting that these genes could illuminate novel pathways for intervention. Thus, our study not only enriches the current understanding of NSOI’s molecular underpinnings but also charts promising avenues for future research and therapeutic innovation in addressing this challenging ocular condition.

Investigations into purine metabolism, a cornerstone for maintaining cellular energy homeostasis and facilitating proliferation, have unearthed its profound implications for various diseases and metabolic pathways. A meticulous literature review highlighted Phosphodiesterase 4B (PDE4B) and Phosphodiesterase 6H (PDE6H) as genes with a potential linkage to NSOI. Drawing upon Zhao’s research, it was observed that reductions in PDE4B and PDE8B levels—enzymes responsible for the catabolic breakdown of cyclic AMP (cAMP)—are associated with the progression of myopia, presumably through the elevation of scleral cAMP concentrations (29). Prior experimental endeavors employing siRNA to diminish PDE4B expression in human scleral fibroblasts resulted in decreased COL1A1 expression, suggesting a regulatory mechanism by PDE4B on scleral collagen I expression dynamics, thereby hinting at PDE4B’s role as a novel susceptibility gene for high myopia (30). Further, PDE6H, alongside five other genes, has been implicated in the etiology and progression of color blindness across diverse ethnic groups, underscoring the critical function of phosphodiesterases (PDEs) in modulating intracellular cyclic nucleotide levels and their vital contributions to various physiological phenomena (31, 32). The focus on PDE4B and PDE6H in relation to ocular diseases has intensified, with research indicating PDE4B’s dysregulation in conditions such as glaucoma, diabetic retinopathy, and age-related macular degeneration (AMD), positing it as a promising therapeutic target. Concurrently, PDE6H’s pivotal role in phototransduction processes within retinal photoreceptor cells has been elucidated, with mutations in PDE6H linked to retinitis pigmentosa, a genetic retinal dystrophy. This body of evidence suggests a nuanced interplay between PDE4B and PDE6H in the modulation of retinal function and intraocular pressure, heralding new insights into ocular disease pathophysiology. Our investigation into the role of these genes within the context of NSOI, supported by dataset GSE105149, proposes a purine metabolism-related trait as a potential prognostic indicator. Despite the burgeoning interest, the exploration of gene alterations associated with purine metabolism in relation to ocular conditions remains scant. The convergence of our findings with existing research underscores the significance of PDE4B and PDE6H as focal points in the landscape of NSOI research, offering promising pathways for therapeutic intervention and a deeper understanding of the molecular intricacies underpinning NSOI.

In the context of NSOI, there’s an emerging discourse suggesting that the amplified immune response isn’t solely tethered to CD4 dynamics. Rather, a nuanced interplay exists involving a pre-established T-regulatory cell landscape, coupled with both proinflammatory and regulatory cascades, such as cytokine disequilibrium (33). This dysregulated immune reconstitution may inadvertently hone in on opportunistic infections, be they active or latent/historically managed. Infections such as Tuberculosis, Cytomegalovirus, progressive multifocal leukoencephalopathy, manifestations like Kaposi’s sarcoma, and an array of autoimmune conditions might escalate or elude detection (34). Among these, Cytomegalovirus retinitis stands out as the predominant opportunistic infection associated with immune restoration inflammatory ocular disorders, notably Immune Recovery Uveitis (24, 35). Mounting evidence suggests that elevating intracellular cAMP concentrations could offer a strategic avenue to attenuate chronic inflammation. One viable modality to elevate cAMP levels hinges on thwarting its degradation, catalyzing the conception of small molecule PDE4 inhibitors. Clinical alleviation in conditions such as inflammatory bowel disease, atopic dermatitis, and rheumatoid arthritis underscores the potential of PDE4 inhibitors (36, 37). Drawing from our antecedent investigations, we delved into the expression dynamics of PMGs within the immunological milieu. Notably, in the treated cohort, there was pronounced expression in naive B cells, memory B cells, follicular helper T cells, resting NK cells, M0 macrophages, and activated mast cells. Conversely, the control ensemble manifested heightened levels of memory cells, activated NK cells, M2 macrophages, and resting mast cells. NSOI pose significant therapeutic challenges, prompting the exploration of novel treatment modalities. Bioinformatics methodologies have emerged as indispensable tools in deciphering the intricate molecular landscape underlying NSOI pathogenesis. Through bioinformatics validation, aberrant gene expression signatures associated with dysregulated immune responses have been delineated in NSOI patients, offering pivotal insights into disease mechanisms. Leveraging these discoveries, investigations into immunotherapeutic interventions targeting these dysregulated pathways have garnered attention. Immunomodulatory agents have demonstrated promise in ameliorating NSOI symptoms by modulating the aberrant immune responses identified through bioinformatics analyses. Furthermore, elucidating the reciprocal influence of immunotherapy on gene expression patterns has unveiled its potential to mitigate neuroinflammation and foster neuroregeneration, thereby presenting a multifaceted approach to NSOI management. This synergistic interaction between bioinformatics validation and immunotherapeutic strategies not only advances our understanding of NSOI pathophysiology but also holds promise for the development of tailored therapeutic interventions.

The exploration of biomarkers in the context of NSOI has been markedly scant, with the nexus between metabolic processes and ocular diseases only recently beginning to unfurl through bioinformatics analyses (38–40). In this emerging field, Liu et al. have delineated hub genes associated with NSOI using Weighted Gene Coexpression Network Analysis, while Hu et al. have crafted a bioinformatics model for thyroid eye disease, identifying 11 pivotal genes including ATP6V1A and PTGES3 among others. Furthermore, Huang et al. have pinpointed six significant genes (CD44, CDC42, TIMP1, BMP7, RHOC, FLT1) as crucial for diabetic retinopathy through advanced bioinformatics analysis coupled with *in vivo* validation. Notwithstanding these advancements, the interconnection between Purine Metabolism and NSOI remains an uncharted territory. Our research endeavors to bridge this gap by delving into cell metabolism, aiming to delineate novel therapeutic strategies for NSOI through the analysis of PMGs extracted from GEO datasets. This novel approach sets our study apart from preceding works, offering fresh theoretical perspectives and methodological innovations. However, our study is not without its limitations. There exists a palpable need for a deeper comprehension of the molecular dynamics that intertwine PMGs with NSOI. The potential for both *in vivo* and *in vitro* studies to shed light on these intricacies is immense, suggesting a plethora of avenues for future inquiry. Moreover, the correlation between prognostic genes and PMGs in the context of NSOI warrants further investigation. Unraveling this intricate relationship could provide invaluable insights into the contributions of PMGs to the pathogenesis and prognosis of NSOI. Such explorations are poised to broaden our understanding and open new therapeutic vistas for the management of NSOI, heralding a future of enhanced insights and interventions in this domain.

## 5 Conclusions

The pathogenesis and progression of NSOI are the result of complex, multifactorial interactions encompassing a multitude of targets, pathways, signaling entities, and regulatory frameworks. These components engage in a synergistic and reciprocal dance that underlies the condition’s intricate nature. Central to this biological interplay are the PMGs, which are crucial in the biosynthesis of a series of proteins including ENTPD1, POLR2K, NPR2, PDE6D, PDE6H, PDE4B, and ALLC. Of particular note, PDE4B and PDE6H are underscored for their prominent roles. Through their activity, they hold the capacity to exert significant influence on the metabolic circuitry, with implications that extend beyond mere biochemical pathways, potentially impacting the clinical course and therapeutic targets in NSOI.

## Data availability statement

The datasets presented in this study can be found in online repositories. The names of the repository/repositories and accession number(s) can be found in the article/**Supplementary Material**.

## Ethics statement

Ethical review and approval was not required as per local legislation and institutional requirements.

## Author contributions

ZW: Conceptualization, Data curation, Writing – original draft. CF: Writing – original draft, Data curation, Formal analysis, Project administration. YH: Investigation, Methodology, Writing – original draft. XP: Investigation, Software, Writing – original draft. ZZ: Formal analysis, Writing – original draft. XY: Conceptualization, Data curation, Methodology, Writing – review & editing. QP: Conceptualization, Investigation, Methodology, Writing – review & editing.
