# Supplementary material for: Bioinformatic validation and machine learning-based exploration of purine metabolism-related gene signatures in the context of immunotherapeutic strategies for nonspecific orbital inflammation
Source: Front Immunol. 2024 Mar 28;15:1318316. doi: 10.3389/fimmu.2024.1318316 (PMC11007227; doi:10.3389/fimmu.2024.1318316)
Supplement: Supplementary file 1 [file DataSheet_1.doc]

**Bioinformatic Validation and Machine Learning-Based Exploration of Purine Metabolism-Related Gene Signatures in the Context of Immunotherapeutic Strategies for Nonspecific Orbital Inflammation**

**Supplementary appendix to the manuscript**

Contents of supplementary appendix

[Appendix 1 3](#__RefHeading___Toc22127)

[Datasets and Purine Metabolism 3](#__RefHeading___Toc32236)

[Table S1. Purine Metabolism Genes 3](#__RefHeading___Toc17747)

[Appendix 2 5](#__RefHeading___Toc4280)

[DEGs linked to PMGs 5](#__RefHeading___Toc30765)

[Table S2. 92 DEGs linked to PMGs. 5](#__RefHeading___Toc6542)

[Appendix 3 9](#__RefHeading___Toc20509)

[Table S3a. Analysis of GO. 9](#__RefHeading___Toc13965)

[Table S3b. Analysis of KEGG. 46](#__RefHeading___Toc5717)

[Appendix 4 48](#__RefHeading___Toc26983)

[Table S4a. LASSO genes. 48](#__RefHeading___Toc21986)

[Table S4b. SVM-RFE genes. 48](#__RefHeading___Toc26766)

[Table S4c. InterGenes. 48](#__RefHeading___Toc19614)

[Appendix 5 49](#__RefHeading___Toc7903)

[Table 5a. PDE4B of GSEA analysis. 49](#__RefHeading___Toc29881)

[Table 5b. PDE6H of GSEA analysis. 57](#__RefHeading___Toc30088)

[Appendix 6 63](#__RefHeading___Toc29561)

[Table 6. Drug prediction. 63](#__RefHeading___Toc8763)

[Appendix 7 65](#__RefHeading___Toc24361)

[Table 7a. Gene-miRNA. 65](#__RefHeading___Toc17393)

[Table 7b. Gene-miRNA. 74](#__RefHeading___Toc7502)

# Appendix 1

**Datasets and Purine Metabolism**

**Table S1. Purine Metabolism Genes**

| NUDT9 | POLA1 | ZNRD1 | AK2 | ENTPD4 | PDE6B |
| --- | --- | --- | --- | --- | --- |
| ADPRM | POLA2 | TWISTNB | AK1 | ENTPD5 | PDE6C |
| NUDT5 | PRIM1 | POLR1E | PDE6A | ENTPD6 | PDE6D |
| PGM1 | PRIM2 | POLR2A | AK8 | NUDT16 | PDE6G |
| PGM2 | POLD1 | POLR2B | AK6 | ITPA | PDE6H |
| PRPS1L1 | POLD2 | POLR2C | AK3 | XDH | PDE9A |
| PRPS2 | POLD3 | POLR2D | ENTPD2 | NUDT2 | PDE10A |
| PRPS1 | POLD4 | POLR2E | NTPCR | GMPS | PDE11A |
| PPAT | POLE | POLR2F | PNPT1 | GMPR | ADSSL1 |
| GART | POLE2 | POLR2G | PDE4A | GMPR2 | ADSS |
| PFAS | POLE3 | POLR2H | PDE4B | GDA | AMPD2 |
| PAICS | POLE4 | POLR2I | PDE4C | GUK1 | AMPD3 |
| ADSL | HDDC3 | POLR2L | PDE4D | PKM | AMPD1 |
| ATIC | PRUNE1 | POLR2J | PDE7A | PKLR | ADK |
| APRT | ADCY1 | POLR2J3 | PDE7B | RRM1 | DCK |
| NT5C2 | ADCY2 | POLR2J2 | PDE8B | RRM2B | ADA |
| NT5C1A | ADCY3 | POLR2K | PDE8A | RRM2 | CECR1 |
| NT5C1B | ADCY4 | POLR3A | FHIT | DGUOK | AK7 |
| NT5C | ADCY5 | POLR3B | ENPP4 | POLR1A | AK4 |
| NT5M | ADCY6 | POLR3C | PAPSS2 | POLR1B | AK5 |
| NT5C3A | ADCY7 | POLR3D | PAPSS1 | NME2 | NPR1 |
| NT5C3B | ADCY8 | POLR3E | ENPP1 | NME4 | NPR2 |
| NT5C1B-RDH14 | ADCY9 | RPC5 | ENPP3 | NME1 | PDE1A |
| NT5E | ADCY10 | POLR1C | URAD | NME3 | PDE1B |
| PNP | GUCY1A2 | POLR3K | ALLC | NME1-NME2 | PDE1C |
| HPRT1 | GUCY1A3 | POLR1D | NME7 | AK9 | PDE2A |
| IMPDH1 | GUCY1B3 | POLR3H | GUCY2F | ENTPD3 | PDE3A |
| IMPDH2 | GUCY2C | POLR3GL | POLR3F | ENTPD8 | PDE3B |
| NME6 | GUCY2D | POLR3G | CANT1 | ENTPD1 | PDE5A |

# Appendix 2

## **DEGs linked to PMGs**

**Table S2. 92 DEGs linked to PMGs.**

| Gene | conMean | treatMean | pvalue | Type |
| --- | --- | --- | --- | --- |
| NUDT9 | 5.05421269 | 4.835023627 | 0.038495536 | Down |
| NUDT5 | 4.61609331 | 4.3870004 | 0.013855985 | Down |
| PGM1 | 6.820155034 | 5.11966992 | 4.91E-12 | Down |
| PRPS2 | 3.04223331 | 2.612434227 | 5.31E-06 | Down |
| PRPS1 | 5.548377345 | 5.06067512 | 1.72E-05 | Down |
| PFAS | 4.432362034 | 4.127166293 | 0.033678548 | Down |
| APRT | 6.214047793 | 6.411802733 | 0.044649661 | Up |
| NT5C1A | 3.047209655 | 3.352686053 | 0.000800619 | Up |
| NT5C | 5.808912379 | 6.219311213 | 5.40E-05 | Up |
| NT5C3A | 4.921345931 | 4.55279836 | 0.014425707 | Down |
| NT5C3B | 6.621764414 | 6.242621387 | 6.40E-05 | Down |
| NT5E | 4.744752103 | 4.206689293 | 7.39E-07 | Down |
| PNP | 4.24090869 | 4.718073333 | 0.003029184 | Up |
| HPRT1 | 2.788967759 | 2.57977504 | 0.023275893 | Down |
| IMPDH2 | 7.642241655 | 7.176053747 | 2.58E-05 | Down |
| NME7 | 3.639234862 | 3.4646218 | 0.006561516 | Down |
| NME3 | 6.014180966 | 6.308491893 | 0.017085787 | Up |
| ENTPD1 | 4.668637586 | 5.31819684 | 2.37E-06 | Up |
| ENTPD4 | 5.369549621 | 5.68432104 | 0.013038283 | Up |
| ENTPD5 | 4.148175069 | 3.970016373 | 0.002755663 | Down |
| NUDT16 | 4.806587828 | 4.569728173 | 0.000105271 | Down |
| ITPA | 5.264088414 | 5.545084693 | 0.000854656 | Up |
| XDH | 2.907195931 | 3.23629316 | 0.011891936 | Up |
| GMPS | 4.594893724 | 4.307351027 | 0.006419441 | Down |
| GMPR | 5.176312069 | 5.491688267 | 0.047824034 | Up |
| PKM | 6.704379414 | 7.20427132 | 2.14E-07 | Up |
| PKLR | 3.178052034 | 3.386198707 | 0.002923761 | Up |
| RRM2B | 3.298066586 | 2.888687293 | 0.002064801 | Down |
| RRM2 | 2.334540379 | 3.119682707 | 1.57E-07 | Up |
| TWISTNB | 2.496792793 | 2.268892627 | 0.006854926 | Down |
| POLR1E | 4.639324483 | 4.731887333 | 0.039872894 | Up |
| POLR2C | 6.07429069 | 5.6540924 | 8.57E-07 | Down |
| POLR2E | 7.685144103 | 7.158638027 | 3.47E-09 | Down |
| POLR2G | 6.022851862 | 5.639150613 | 0.001594765 | Down |
| POLR2I | 8.120137862 | 7.47839748 | 8.57E-07 | Down |
| POLR2L | 7.029220586 | 6.79387136 | 0.015166374 | Down |
| POLR2J | 9.254660172 | 8.90958684 | 0.008584609 | Down |
| POLR2J2 | 6.852725586 | 7.54754428 | 7.81E-07 | Up |
| POLR2K | 5.678211966 | 4.733280213 | 2.05E-10 | Down |
| POLR3A | 3.560730034 | 3.698749773 | 0.000502472 | Up |
| POLR3B | 2.666908276 | 2.439488987 | 2.62E-05 | Down |
| POLR3C | 4.509542931 | 4.210439347 | 0.001827105 | Down |
| POLR3D | 4.504003793 | 4.813809827 | 0.000559693 | Up |
| POLR3K | 4.425063621 | 4.749021747 | 0.006633899 | Up |
| POLR1D | 5.351563 | 4.804173173 | 0.001872541 | Down |
| POLR3GL | 5.046153034 | 4.664763187 | 3.54E-05 | Down |
| POLR3F | 2.637410172 | 2.307438253 | 0.00026619 | Down |
| POLA1 | 4.534878724 | 3.919916053 | 2.16E-05 | Down |
| POLA2 | 4.329254793 | 4.645133293 | 2.76E-07 | Up |
| POLD1 | 4.590238034 | 4.991002747 | 0.000178505 | Up |
| POLD2 | 6.680643069 | 6.48555828 | 0.015784051 | Down |
| POLD4 | 6.537320345 | 7.179984173 | 8.27E-09 | Up |
| POLE | 5.775110379 | 6.239795653 | 0.000200285 | Up |
| POLE3 | 3.168624897 | 2.661731653 | 0.001256535 | Down |
| POLE4 | 4.863895724 | 4.428417173 | 1.82E-11 | Down |
| ADCY2 | 3.423157138 | 2.926784787 | 2.13E-06 | Down |
| ADCY5 | 5.131969207 | 4.67297392 | 2.05E-06 | Down |
| ADCY9 | 6.477490138 | 6.249786253 | 0.000523234 | Down |
| GUCY1A2 | 3.541861552 | 2.982097573 | 2.02E-07 | Down |
| GUCY1A3 | 5.956493552 | 5.213212187 | 1.78E-05 | Down |
| GUCY1B3 | 4.607795655 | 4.227703213 | 0.009863224 | Down |
| GUCY2C | 3.335766517 | 3.551361013 | 0.033678548 | Up |
| GUCY2D | 3.450829172 | 3.82949844 | 0.010723394 | Up |
| NPR1 | 6.585051897 | 6.168868653 | 0.002958498 | Down |
| NPR2 | 5.624283103 | 4.77783276 | 3.32E-08 | Down |
| PDE1A | 2.519477552 | 2.307250027 | 0.003486012 | Down |
| PDE2A | 5.996355655 | 5.091887587 | 1.33E-06 | Down |
| PDE3B | 4.432020759 | 3.313995867 | 5.25E-09 | Down |
| PDE5A | 4.682515414 | 4.12839576 | 1.84E-05 | Down |
| PDE6A | 4.424120793 | 4.7491408 | 0.001536311 | Up |
| PDE6B | 4.168934207 | 4.46392948 | 0.000206126 | Up |
| PDE6D | 4.398023448 | 4.226581413 | 0.005376789 | Down |
| PDE6G | 5.224232241 | 5.648348493 | 6.90E-05 | Up |
| PDE6H | 1.747199793 | 1.614790347 | 0.045035689 | Down |
| AMPD2 | 5.593527276 | 5.860596813 | 0.019972398 | Up |
| AMPD3 | 5.084410759 | 5.41009204 | 0.014718639 | Up |
| AMPD1 | 3.624853 | 3.868849107 | 0.011178099 | Up |
| ADK | 3.986885897 | 3.685019347 | 0.01290647 | Down |
| ADA | 4.490865724 | 4.73580704 | 0.019213444 | Up |
| CECR1 | 6.51036931 | 7.121433387 | 0.002220807 | Up |
| AK5 | 4.140351207 | 4.266238773 | 0.048646762 | Up |
| AK8 | 3.976578586 | 4.202527707 | 0.011649622 | Up |
| AK6 | 5.460137586 | 4.89742372 | 0.001536356 | Down |
| AK3 | 5.253386828 | 4.36220236 | 2.83E-06 | Down |
| ENTPD2 | 5.029697241 | 5.360797387 | 0.002275081 | Up |
| NTPCR | 4.731741586 | 4.055958173 | 2.79E-12 | Down |
| PDE4B | 3.767877241 | 4.399775027 | 0.000832674 | Up |
| PDE7A | 3.475385 | 4.045695293 | 7.21E-08 | Up |
| ENPP4 | 2.410338172 | 2.109065133 | 0.003327257 | Down |
| PAPSS2 | 4.585260448 | 4.079071147 | 0.001782684 | Down |
| ENPP1 | 3.563907207 | 3.146541547 | 6.30E-05 | Down |
| ALLC | 2.616395448 | 2.917917573 | 0.0027885 | Up |

# Appendix 3

**Analysis of enrichment**

**Table S3a. Analysis of GO.**

| ONTOLOGY | ID | Description | BgRatio | pvalue | qvalue | Count |
| --- | --- | --- | --- | --- | --- | --- |
| BP | GO:0072521 | purine-containing compound metabolic process | 460/18862 | 3.12E-51 | 2.29E-48 | 46 |
| BP | GO:0006163 | purine nucleotide metabolic process | 441/18862 | 2.14E-50 | 7.85E-48 | 45 |
| BP | GO:0019693 | ribose phosphate metabolic process | 435/18862 | 1.17E-45 | 2.86E-43 | 42 |
| BP | GO:0009150 | purine ribonucleotide metabolic process | 408/18862 | 1.47E-43 | 2.70E-41 | 40 |
| BP | GO:0009259 | ribonucleotide metabolic process | 425/18862 | 7.85E-43 | 1.15E-40 | 40 |
| BP | GO:0034404 | nucleobase-containing small molecule biosynthetic process | 115/18862 | 1.04E-41 | 1.27E-39 | 28 |
| BP | GO:0009165 | nucleotide biosynthetic process | 264/18862 | 2.22E-37 | 2.27E-35 | 32 |
| BP | GO:0072522 | purine-containing compound biosynthetic process | 208/18862 | 2.48E-37 | 2.27E-35 | 30 |
| BP | GO:1901293 | nucleoside phosphate biosynthetic process | 267/18862 | 3.23E-37 | 2.63E-35 | 32 |
| BP | GO:1901292 | nucleoside phosphate catabolic process | 83/18862 | 1.26E-33 | 9.28E-32 | 22 |
| BP | GO:0006164 | purine nucleotide biosynthetic process | 197/18862 | 7.00E-33 | 4.67E-31 | 27 |
| BP | GO:0046390 | ribose phosphate biosynthetic process | 195/18862 | 2.56E-31 | 1.56E-29 | 26 |
| BP | GO:0009152 | purine ribonucleotide biosynthetic process | 175/18862 | 6.91E-31 | 3.90E-29 | 25 |
| BP | GO:0009123 | nucleoside monophosphate metabolic process | 75/18862 | 1.20E-30 | 6.27E-29 | 20 |
| BP | GO:0072523 | purine-containing compound catabolic process | 51/18862 | 3.38E-30 | 1.65E-28 | 18 |
| BP | GO:0009260 | ribonucleotide biosynthetic process | 188/18862 | 4.53E-30 | 2.08E-28 | 25 |
| BP | GO:0006195 | purine nucleotide catabolic process | 46/18862 | 5.78E-29 | 2.50E-27 | 17 |
| BP | GO:0009126 | purine nucleoside monophosphate metabolic process | 43/18862 | 2.34E-27 | 9.54E-26 | 16 |
| BP | GO:0046434 | organophosphate catabolic process | 154/18862 | 3.55E-27 | 1.37E-25 | 22 |
| BP | GO:0034656 | nucleobase-containing small molecule catabolic process | 50/18862 | 4.24E-26 | 1.56E-24 | 16 |
| BP | GO:0009167 | purine ribonucleoside monophosphate metabolic process | 40/18862 | 9.35E-26 | 3.27E-24 | 15 |
| BP | GO:0009116 | nucleoside metabolic process | 104/18862 | 1.12E-25 | 3.72E-24 | 19 |
| BP | GO:0009161 | ribonucleoside monophosphate metabolic process | 56/18862 | 3.51E-25 | 1.12E-23 | 16 |
| BP | GO:0009166 | nucleotide catabolic process | 73/18862 | 5.31E-25 | 1.62E-23 | 17 |
| BP | GO:0009154 | purine ribonucleotide catabolic process | 35/18862 | 1.41E-24 | 4.14E-23 | 14 |
| BP | GO:1901657 | glycosyl compound metabolic process | 129/18862 | 8.78E-24 | 2.48E-22 | 19 |
| BP | GO:0009187 | cyclic nucleotide metabolic process | 40/18862 | 1.39E-23 | 3.63E-22 | 14 |
| BP | GO:0009261 | ribonucleotide catabolic process | 40/18862 | 1.39E-23 | 3.63E-22 | 14 |
| BP | GO:0046128 | purine ribonucleoside metabolic process | 58/18862 | 6.48E-23 | 1.64E-21 | 15 |
| BP | GO:0042278 | purine nucleoside metabolic process | 61/18862 | 1.52E-22 | 3.72E-21 | 15 |
| BP | GO:0009119 | ribonucleoside metabolic process | 72/18862 | 2.39E-21 | 5.67E-20 | 15 |
| BP | GO:0009168 | purine ribonucleoside monophosphate biosynthetic process | 21/18862 | 3.54E-21 | 8.13E-20 | 11 |
| BP | GO:0009127 | purine nucleoside monophosphate biosynthetic process | 23/18862 | 1.35E-20 | 3.00E-19 | 11 |
| BP | GO:0009156 | ribonucleoside monophosphate biosynthetic process | 34/18862 | 2.13E-20 | 4.59E-19 | 12 |
| BP | GO:1901136 | carbohydrate derivative catabolic process | 198/18862 | 3.92E-20 | 8.22E-19 | 19 |
| BP | GO:0009124 | nucleoside monophosphate biosynthetic process | 43/18862 | 5.75E-19 | 1.17E-17 | 12 |
| BP | GO:0043101 | purine-containing compound salvage | 15/18862 | 3.02E-18 | 5.99E-17 | 9 |
| BP | GO:0009141 | nucleoside triphosphate metabolic process | 109/18862 | 7.46E-17 | 1.44E-15 | 14 |
| BP | GO:0009190 | cyclic nucleotide biosynthetic process | 22/18862 | 2.92E-16 | 4.99E-15 | 9 |
| BP | GO:0042451 | purine nucleoside biosynthetic process | 22/18862 | 2.92E-16 | 4.99E-15 | 9 |
| BP | GO:0042455 | ribonucleoside biosynthetic process | 22/18862 | 2.92E-16 | 4.99E-15 | 9 |
| BP | GO:0046129 | purine ribonucleoside biosynthetic process | 22/18862 | 2.92E-16 | 4.99E-15 | 9 |
| BP | GO:0052652 | cyclic purine nucleotide metabolic process | 22/18862 | 2.92E-16 | 4.99E-15 | 9 |
| BP | GO:0009164 | nucleoside catabolic process | 34/18862 | 3.06E-16 | 5.11E-15 | 10 |
| BP | GO:0009132 | nucleoside diphosphate metabolic process | 156/18862 | 4.61E-16 | 7.52E-15 | 15 |
| BP | GO:0046033 | AMP metabolic process | 16/18862 | 1.84E-15 | 2.93E-14 | 8 |
| BP | GO:0046068 | cGMP metabolic process | 17/18862 | 3.46E-15 | 5.40E-14 | 8 |
| BP | GO:1901658 | glycosyl compound catabolic process | 44/18862 | 5.58E-15 | 8.53E-14 | 10 |
| BP | GO:0009134 | nucleoside diphosphate catabolic process | 12/18862 | 2.70E-14 | 4.04E-13 | 7 |
| BP | GO:0043094 | cellular metabolic compound salvage | 34/18862 | 2.95E-14 | 4.33E-13 | 9 |
| BP | GO:0009125 | nucleoside monophosphate catabolic process | 13/18862 | 5.82E-14 | 8.38E-13 | 7 |
| BP | GO:0044282 | small molecule catabolic process | 431/18862 | 7.46E-14 | 1.05E-12 | 19 |
| BP | GO:0009163 | nucleoside biosynthetic process | 38/18862 | 9.03E-14 | 1.25E-12 | 9 |
| BP | GO:0043173 | nucleotide salvage | 14/18862 | 1.16E-13 | 1.58E-12 | 7 |
| BP | GO:1901659 | glycosyl compound biosynthetic process | 42/18862 | 2.43E-13 | 3.25E-12 | 9 |
| BP | GO:0032201 | telomere maintenance via semi-conservative replication | 27/18862 | 3.05E-13 | 3.99E-12 | 8 |
| BP | GO:0006283 | transcription-coupled nucleotide-excision repair | 73/18862 | 1.25E-12 | 1.62E-11 | 10 |
| BP | GO:0006152 | purine nucleoside catabolic process | 10/18862 | 1.67E-12 | 2.05E-11 | 6 |
| BP | GO:0046130 | purine ribonucleoside catabolic process | 10/18862 | 1.67E-12 | 2.05E-11 | 6 |
| BP | GO:0006144 | purine nucleobase metabolic process | 19/18862 | 1.67E-12 | 2.05E-11 | 7 |
| BP | GO:0032481 | positive regulation of type I interferon production | 77/18862 | 2.18E-12 | 2.63E-11 | 10 |
| BP | GO:0006289 | nucleotide-excision repair | 108/18862 | 2.51E-12 | 2.97E-11 | 11 |
| BP | GO:0072527 | pyrimidine-containing compound metabolic process | 85/18862 | 6.04E-12 | 7.04E-11 | 10 |
| BP | GO:0009144 | purine nucleoside triphosphate metabolic process | 86/18862 | 6.81E-12 | 7.81E-11 | 10 |
| BP | GO:0009262 | deoxyribonucleotide metabolic process | 40/18862 | 1.01E-11 | 1.12E-10 | 8 |
| BP | GO:1901068 | guanosine-containing compound metabolic process | 40/18862 | 1.01E-11 | 1.12E-10 | 8 |
| BP | GO:0032479 | regulation of type I interferon production | 128/18862 | 1.64E-11 | 1.80E-10 | 11 |
| BP | GO:0032606 | type I interferon production | 129/18862 | 1.79E-11 | 1.93E-10 | 11 |
| BP | GO:0050434 | positive regulation of viral transcription | 26/18862 | 2.13E-11 | 2.26E-10 | 7 |
| BP | GO:0009151 | purine deoxyribonucleotide metabolic process | 14/18862 | 2.36E-11 | 2.43E-10 | 6 |
| BP | GO:0046040 | IMP metabolic process | 14/18862 | 2.36E-11 | 2.43E-10 | 6 |
| BP | GO:0009185 | ribonucleoside diphosphate metabolic process | 138/18862 | 3.75E-11 | 3.82E-10 | 11 |
| BP | GO:0006370 | 7-methylguanosine mRNA capping | 33/18862 | 1.35E-10 | 1.35E-09 | 7 |
| BP | GO:0009112 | nucleobase metabolic process | 34/18862 | 1.69E-10 | 1.63E-09 | 7 |
| BP | GO:0009452 | 7-methylguanosine RNA capping | 34/18862 | 1.69E-10 | 1.63E-09 | 7 |
| BP | GO:0036260 | RNA capping | 34/18862 | 1.69E-10 | 1.63E-09 | 7 |
| BP | GO:0009142 | nucleoside triphosphate biosynthetic process | 84/18862 | 1.72E-10 | 1.64E-09 | 9 |
| BP | GO:0033260 | nuclear DNA replication | 59/18862 | 2.70E-10 | 2.54E-09 | 8 |
| BP | GO:0006188 | IMP biosynthetic process | 10/18862 | 4.60E-10 | 4.22E-09 | 5 |
| BP | GO:0009113 | purine nucleobase biosynthetic process | 10/18862 | 4.60E-10 | 4.22E-09 | 5 |
| BP | GO:0044786 | cell cycle DNA replication | 64/18862 | 5.29E-10 | 4.79E-09 | 8 |
| BP | GO:0009135 | purine nucleoside diphosphate metabolic process | 135/18862 | 6.31E-10 | 5.58E-09 | 10 |
| BP | GO:0009179 | purine ribonucleoside diphosphate metabolic process | 135/18862 | 6.31E-10 | 5.58E-09 | 10 |
| BP | GO:0046782 | regulation of viral transcription | 42/18862 | 8.22E-10 | 7.18E-09 | 7 |
| BP | GO:0006182 | cGMP biosynthetic process | 11/18862 | 8.40E-10 | 7.25E-09 | 5 |
| BP | GO:0042454 | ribonucleoside catabolic process | 24/18862 | 1.02E-09 | 8.68E-09 | 6 |
| BP | GO:0006383 | transcription by RNA polymerase III | 47/18862 | 1.88E-09 | 1.59E-08 | 7 |
| BP | GO:0015949 | nucleobase-containing small molecule interconversion | 27/18862 | 2.21E-09 | 1.85E-08 | 6 |
| BP | GO:1901070 | guanosine-containing compound biosynthetic process | 13/18862 | 2.32E-09 | 1.92E-08 | 5 |
| BP | GO:0006220 | pyrimidine nucleotide metabolic process | 50/18862 | 2.95E-09 | 2.41E-08 | 7 |
| BP | GO:0009218 | pyrimidine ribonucleotide metabolic process | 29/18862 | 3.53E-09 | 2.84E-08 | 6 |
| BP | GO:0009214 | cyclic nucleotide catabolic process | 14/18862 | 3.60E-09 | 2.87E-08 | 5 |
| BP | GO:0009199 | ribonucleoside triphosphate metabolic process | 87/18862 | 6.41E-09 | 5.06E-08 | 8 |
| BP | GO:0009394 | 2'-deoxyribonucleotide metabolic process | 36/18862 | 1.41E-08 | 1.10E-07 | 6 |
| BP | GO:0046112 | nucleobase biosynthetic process | 18/18862 | 1.52E-08 | 1.17E-07 | 5 |
| BP | GO:0019692 | deoxyribose phosphate metabolic process | 38/18862 | 1.98E-08 | 1.52E-07 | 6 |
| BP | GO:0035019 | somatic stem cell population maintenance | 71/18862 | 3.64E-08 | 2.75E-07 | 7 |
| BP | GO:0098781 | ncRNA transcription | 109/18862 | 3.84E-08 | 2.87E-07 | 8 |
| BP | GO:0046058 | cAMP metabolic process | 22/18862 | 4.60E-08 | 3.41E-07 | 5 |
| BP | GO:0042795 | snRNA transcription by RNA polymerase II | 74/18862 | 4.87E-08 | 3.57E-07 | 7 |
| BP | GO:0009301 | snRNA transcription | 75/18862 | 5.35E-08 | 3.89E-07 | 7 |
| BP | GO:0006354 | DNA-templated transcription, elongation | 119/18862 | 7.63E-08 | 5.49E-07 | 8 |
| BP | GO:0009205 | purine ribonucleoside triphosphate metabolic process | 80/18862 | 8.41E-08 | 5.99E-07 | 7 |
| BP | GO:0007168 | receptor guanylyl cyclase signaling pathway | 10/18862 | 8.68E-08 | 6.12E-07 | 4 |
| BP | GO:0006165 | nucleoside diphosphate phosphorylation | 132/18862 | 1.71E-07 | 1.19E-06 | 8 |
| BP | GO:0046939 | nucleotide phosphorylation | 133/18862 | 1.81E-07 | 1.25E-06 | 8 |
| BP | GO:0006368 | transcription elongation from RNA polymerase II promoter | 90/18862 | 1.90E-07 | 1.31E-06 | 7 |
| BP | GO:0048524 | positive regulation of viral process | 91/18862 | 2.06E-07 | 1.40E-06 | 7 |
| BP | GO:0046940 | nucleoside monophosphate phosphorylation | 13/18862 | 2.92E-07 | 1.97E-06 | 4 |
| BP | GO:0032728 | positive regulation of interferon-beta production | 33/18862 | 3.98E-07 | 2.66E-06 | 5 |
| BP | GO:0009143 | nucleoside triphosphate catabolic process | 14/18862 | 4.08E-07 | 2.70E-06 | 4 |
| BP | GO:0009263 | deoxyribonucleotide biosynthetic process | 15/18862 | 5.54E-07 | 3.60E-06 | 4 |
| BP | GO:0046037 | GMP metabolic process | 15/18862 | 5.54E-07 | 3.60E-06 | 4 |
| BP | GO:0006261 | DNA-dependent DNA replication | 157/18862 | 6.45E-07 | 4.13E-06 | 8 |
| BP | GO:0006260 | DNA replication | 280/18862 | 6.47E-07 | 4.13E-06 | 10 |
| BP | GO:0009145 | purine nucleoside triphosphate biosynthetic process | 68/18862 | 7.04E-07 | 4.45E-06 | 6 |
| BP | GO:0009200 | deoxyribonucleoside triphosphate metabolic process | 16/18862 | 7.37E-07 | 4.62E-06 | 4 |
| BP | GO:0000723 | telomere maintenance | 161/18862 | 7.81E-07 | 4.85E-06 | 8 |
| BP | GO:0008543 | fibroblast growth factor receptor signaling pathway | 113/18862 | 9.04E-07 | 5.57E-06 | 7 |
| BP | GO:0006270 | DNA replication initiation | 40/18862 | 1.08E-06 | 6.53E-06 | 5 |
| BP | GO:0072529 | pyrimidine-containing compound catabolic process | 40/18862 | 1.08E-06 | 6.53E-06 | 5 |
| BP | GO:0009208 | pyrimidine ribonucleoside triphosphate metabolic process | 18/18862 | 1.23E-06 | 7.40E-06 | 4 |
| BP | GO:0032200 | telomere organization | 174/18862 | 1.40E-06 | 8.37E-06 | 8 |
| BP | GO:0060964 | regulation of gene silencing by miRNA | 121/18862 | 1.43E-06 | 8.49E-06 | 7 |
| BP | GO:0046031 | ADP metabolic process | 122/18862 | 1.52E-06 | 8.90E-06 | 7 |
| BP | GO:0006271 | DNA strand elongation involved in DNA replication | 19/18862 | 1.55E-06 | 9.04E-06 | 4 |
| BP | GO:0060147 | regulation of posttranscriptional gene silencing | 124/18862 | 1.69E-06 | 9.77E-06 | 7 |
| BP | GO:0060966 | regulation of gene silencing by RNA | 125/18862 | 1.79E-06 | 1.02E-05 | 7 |
| BP | GO:0032648 | regulation of interferon-beta production | 50/18862 | 3.34E-06 | 1.90E-05 | 5 |
| BP | GO:0019935 | cyclic-nucleotide-mediated signaling | 89/18862 | 3.46E-06 | 1.94E-05 | 6 |
| BP | GO:0006297 | nucleotide-excision repair, DNA gap filling | 23/18862 | 3.50E-06 | 1.94E-05 | 4 |
| BP | GO:0046135 | pyrimidine nucleoside catabolic process | 23/18862 | 3.50E-06 | 1.94E-05 | 4 |
| BP | GO:0032608 | interferon-beta production | 52/18862 | 4.07E-06 | 2.25E-05 | 5 |
| BP | GO:0044344 | cellular response to fibroblast growth factor stimulus | 142/18862 | 4.18E-06 | 2.27E-05 | 7 |
| BP | GO:0046039 | GTP metabolic process | 24/18862 | 4.18E-06 | 2.27E-05 | 4 |
| BP | GO:0000731 | DNA synthesis involved in DNA repair | 53/18862 | 4.48E-06 | 2.42E-05 | 5 |
| BP | GO:0008277 | regulation of G protein-coupled receptor signaling pathway | 144/18862 | 4.58E-06 | 2.44E-05 | 7 |
| BP | GO:0060968 | regulation of gene silencing | 144/18862 | 4.58E-06 | 2.44E-05 | 7 |
| BP | GO:0009147 | pyrimidine nucleoside triphosphate metabolic process | 25/18862 | 4.96E-06 | 2.62E-05 | 4 |
| BP | GO:0019827 | stem cell population maintenance | 146/18862 | 5.02E-06 | 2.63E-05 | 7 |
| BP | GO:0001819 | positive regulation of cytokine production | 437/18862 | 5.24E-06 | 2.73E-05 | 11 |
| BP | GO:0071774 | response to fibroblast growth factor | 148/18862 | 5.49E-06 | 2.82E-05 | 7 |
| BP | GO:0098727 | maintenance of cell number | 148/18862 | 5.49E-06 | 2.82E-05 | 7 |
| BP | GO:0009264 | deoxyribonucleotide catabolic process | 26/18862 | 5.84E-06 | 2.96E-05 | 4 |
| BP | GO:0022616 | DNA strand elongation | 26/18862 | 5.84E-06 | 2.96E-05 | 4 |
| BP | GO:0046148 | pigment biosynthetic process | 57/18862 | 6.44E-06 | 3.24E-05 | 5 |
| BP | GO:0022400 | regulation of rhodopsin mediated signaling pathway | 27/18862 | 6.83E-06 | 3.39E-05 | 4 |
| BP | GO:0046386 | deoxyribose phosphate catabolic process | 27/18862 | 6.83E-06 | 3.39E-05 | 4 |
| BP | GO:0006221 | pyrimidine nucleotide biosynthetic process | 30/18862 | 1.06E-05 | 5.13E-05 | 4 |
| BP | GO:0006362 | transcription elongation from RNA polymerase I promoter | 30/18862 | 1.06E-05 | 5.13E-05 | 4 |
| BP | GO:0016056 | rhodopsin mediated signaling pathway | 30/18862 | 1.06E-05 | 5.13E-05 | 4 |
| BP | GO:0006171 | cAMP biosynthetic process | 10/18862 | 1.11E-05 | 5.36E-05 | 3 |
| BP | GO:0006363 | termination of RNA polymerase I transcription | 31/18862 | 1.21E-05 | 5.79E-05 | 4 |
| BP | GO:0009206 | purine ribonucleoside triphosphate biosynthetic process | 67/18862 | 1.43E-05 | 6.83E-05 | 5 |
| BP | GO:0006183 | GTP biosynthetic process | 11/18862 | 1.52E-05 | 7.21E-05 | 3 |
| BP | GO:0006352 | DNA-templated transcription, initiation | 249/18862 | 1.96E-05 | 9.20E-05 | 8 |
| BP | GO:0019083 | viral transcription | 180/18862 | 1.97E-05 | 9.20E-05 | 7 |
| BP | GO:0007603 | phototransduction, visible light | 35/18862 | 1.98E-05 | 9.20E-05 | 4 |
| BP | GO:0046051 | UTP metabolic process | 12/18862 | 2.02E-05 | 9.34E-05 | 3 |
| BP | GO:0042440 | pigment metabolic process | 72/18862 | 2.04E-05 | 9.35E-05 | 5 |
| BP | GO:0009201 | ribonucleoside triphosphate biosynthetic process | 73/18862 | 2.18E-05 | 9.94E-05 | 5 |
| BP | GO:0006213 | pyrimidine nucleoside metabolic process | 36/18862 | 2.22E-05 | 0.000100626 | 4 |
| BP | GO:0050792 | regulation of viral process | 186/18862 | 2.43E-05 | 0.00010954 | 7 |
| BP | GO:0006353 | DNA-templated transcription, termination | 75/18862 | 2.49E-05 | 0.000111338 | 5 |
| BP | GO:0006367 | transcription initiation from RNA polymerase II promoter | 187/18862 | 2.52E-05 | 0.000112004 | 7 |
| BP | GO:0006361 | transcription initiation from RNA polymerase I promoter | 38/18862 | 2.76E-05 | 0.000122195 | 4 |
| BP | GO:0071897 | DNA biosynthetic process | 194/18862 | 3.19E-05 | 0.000140039 | 7 |
| BP | GO:0010752 | regulation of cGMP-mediated signaling | 14/18862 | 3.33E-05 | 0.000145236 | 3 |
| BP | GO:0072528 | pyrimidine-containing compound biosynthetic process | 40/18862 | 3.40E-05 | 0.000147563 | 4 |
| BP | GO:0043903 | regulation of biological process involved in symbiotic interaction | 197/18862 | 3.52E-05 | 0.00015173 | 7 |
| BP | GO:0019080 | viral gene expression | 198/18862 | 3.63E-05 | 0.000155792 | 7 |
| BP | GO:0043174 | nucleoside salvage | 16/18862 | 5.08E-05 | 0.000215537 | 3 |
| BP | GO:0046036 | CTP metabolic process | 16/18862 | 5.08E-05 | 0.000215537 | 3 |
| BP | GO:0009162 | deoxyribonucleoside monophosphate metabolic process | 17/18862 | 6.15E-05 | 0.000259352 | 3 |
| BP | GO:0034199 | activation of protein kinase A activity | 18/18862 | 7.36E-05 | 0.000308413 | 3 |
| BP | GO:0090305 | nucleic acid phosphodiester bond hydrolysis | 305/18862 | 8.24E-05 | 0.000343313 | 8 |
| BP | GO:0019932 | second-messenger-mediated signaling | 307/18862 | 8.62E-05 | 0.000357244 | 8 |
| BP | GO:0071375 | cellular response to peptide hormone stimulus | 325/18862 | 0.000127789 | 0.000526723 | 8 |
| BP | GO:0009220 | pyrimidine ribonucleotide biosynthetic process | 22/18862 | 0.000136993 | 0.000561505 | 3 |
| BP | GO:0045815 | positive regulation of gene expression, epigenetic | 58/18862 | 0.000148134 | 0.0006038 | 4 |
| BP | GO:0007602 | phototransduction | 59/18862 | 0.000158352 | 0.000641879 | 4 |
| BP | GO:0019933 | cAMP-mediated signaling | 62/18862 | 0.00019206 | 0.000770006 | 4 |
| BP | GO:0046365 | monosaccharide catabolic process | 62/18862 | 0.00019206 | 0.000770006 | 4 |
| BP | GO:0051607 | defense response to virus | 260/18862 | 0.000199358 | 0.000790625 | 7 |
| BP | GO:0140546 | defense response to symbiont | 260/18862 | 0.000199358 | 0.000790625 | 7 |
| BP | GO:0071377 | cellular response to glucagon stimulus | 26/18862 | 0.000228223 | 0.000900233 | 3 |
| BP | GO:0009584 | detection of visible light | 66/18862 | 0.000244681 | 0.000959994 | 4 |
| BP | GO:0006360 | transcription by RNA polymerase I | 68/18862 | 0.00027455 | 0.001071451 | 4 |
| BP | GO:0019934 | cGMP-mediated signaling | 30/18862 | 0.000351662 | 0.001365124 | 3 |
| BP | GO:0071482 | cellular response to light stimulus | 134/18862 | 0.000389117 | 0.001502573 | 5 |
| BP | GO:0009583 | detection of light stimulus | 77/18862 | 0.000442071 | 0.001698117 | 4 |
| BP | GO:1901653 | cellular response to peptide | 391/18862 | 0.000445156 | 0.001701062 | 8 |
| BP | GO:0007601 | visual perception | 215/18862 | 0.000479264 | 0.001821911 | 6 |
| BP | GO:0050953 | sensory perception of light stimulus | 219/18862 | 0.000528221 | 0.001997666 | 6 |
| BP | GO:0046034 | ATP metabolic process | 313/18862 | 0.000608854 | 0.002290805 | 7 |
| BP | GO:0007193 | adenylate cyclase-inhibiting G protein-coupled receptor signaling pathway | 85/18862 | 0.000643179 | 0.002407602 | 4 |
| BP | GO:0006296 | nucleotide-excision repair, DNA incision, 5'-to lesion | 37/18862 | 0.000657512 | 0.002436394 | 3 |
| BP | GO:0033762 | response to glucagon | 37/18862 | 0.000657512 | 0.002436394 | 3 |
| BP | GO:0007589 | body fluid secretion | 86/18862 | 0.000672199 | 0.0024783 | 4 |
| BP | GO:0003091 | renal water homeostasis | 38/18862 | 0.0007115 | 0.00258424 | 3 |
| BP | GO:0006298 | mismatch repair | 38/18862 | 0.0007115 | 0.00258424 | 3 |
| BP | GO:0007223 | Wnt signaling pathway, calcium modulating pathway | 38/18862 | 0.0007115 | 0.00258424 | 3 |
| BP | GO:0007190 | activation of adenylate cyclase activity | 39/18862 | 0.000768234 | 0.002749468 | 3 |
| BP | GO:0033683 | nucleotide-excision repair, DNA incision | 39/18862 | 0.000768234 | 0.002749468 | 3 |
| BP | GO:0042769 | DNA damage response, detection of DNA damage | 39/18862 | 0.000768234 | 0.002749468 | 3 |
| BP | GO:0043434 | response to peptide hormone | 435/18862 | 0.000893509 | 0.003182299 | 8 |
| BP | GO:0006228 | UTP biosynthetic process | 10/18862 | 0.000923922 | 0.003227939 | 2 |
| BP | GO:0009133 | nucleoside diphosphate biosynthetic process | 10/18862 | 0.000923922 | 0.003227939 | 2 |
| BP | GO:1904321 | response to forskolin | 10/18862 | 0.000923922 | 0.003227939 | 2 |
| BP | GO:1904322 | cellular response to forskolin | 10/18862 | 0.000923922 | 0.003227939 | 2 |
| BP | GO:0019985 | translesion synthesis | 42/18862 | 0.000955447 | 0.003322259 | 3 |
| BP | GO:0001973 | G protein-coupled adenosine receptor signaling pathway | 12/18862 | 0.001346978 | 0.004575098 | 2 |
| BP | GO:0009265 | 2'-deoxyribonucleotide biosynthetic process | 12/18862 | 0.001346978 | 0.004575098 | 2 |
| BP | GO:0035588 | G protein-coupled purinergic receptor signaling pathway | 12/18862 | 0.001346978 | 0.004575098 | 2 |
| BP | GO:0046385 | deoxyribose phosphate biosynthetic process | 12/18862 | 0.001346978 | 0.004575098 | 2 |
| BP | GO:0071872 | cellular response to epinephrine stimulus | 12/18862 | 0.001346978 | 0.004575098 | 2 |
| BP | GO:0009615 | response to virus | 359/18862 | 0.001353166 | 0.004575098 | 7 |
| BP | GO:0060249 | anatomical structure homeostasis | 466/18862 | 0.001387057 | 0.004668172 | 8 |
| BP | GO:0019674 | NAD metabolic process | 51/18862 | 0.001682191 | 0.005635603 | 3 |
| BP | GO:0000082 | G1/S transition of mitotic cell cycle | 275/18862 | 0.001711722 | 0.005708469 | 6 |
| BP | GO:0006301 | postreplication repair | 52/18862 | 0.001779272 | 0.005906896 | 3 |
| BP | GO:0006241 | CTP biosynthetic process | 14/18862 | 0.001846094 | 0.006046651 | 2 |
| BP | GO:0043951 | negative regulation of cAMP-mediated signaling | 14/18862 | 0.001846094 | 0.006046651 | 2 |
| BP | GO:0046415 | urate metabolic process | 14/18862 | 0.001846094 | 0.006046651 | 2 |
| BP | GO:0071478 | cellular response to radiation | 191/18862 | 0.001908331 | 0.006218803 | 5 |
| BP | GO:0006096 | glycolytic process | 114/18862 | 0.001915605 | 0.006218803 | 4 |
| BP | GO:0000377 | RNA splicing, via transesterification reactions with bulged adenosine as nucleophile | 383/18862 | 0.001955387 | 0.006292265 | 7 |
| BP | GO:0000398 | mRNA splicing, via spliceosome | 383/18862 | 0.001955387 | 0.006292265 | 7 |
| BP | GO:0006757 | ATP generation from ADP | 115/18862 | 0.001977733 | 0.006336383 | 4 |
| BP | GO:0000375 | RNA splicing, via transesterification reactions | 386/18862 | 0.00204331 | 0.00651802 | 7 |
| BP | GO:0009209 | pyrimidine ribonucleoside triphosphate biosynthetic process | 15/18862 | 0.002123734 | 0.006716164 | 2 |
| BP | GO:0071871 | response to epinephrine | 15/18862 | 0.002123734 | 0.006716164 | 2 |
| BP | GO:0003014 | renal system process | 118/18862 | 0.002172471 | 0.006840805 | 4 |
| BP | GO:0016052 | carbohydrate catabolic process | 197/18862 | 0.00218365 | 0.006846622 | 5 |
| BP | GO:0019320 | hexose catabolic process | 56/18862 | 0.002202344 | 0.006875852 | 3 |
| BP | GO:0007625 | grooming behavior | 16/18862 | 0.002419864 | 0.007522949 | 2 |
| BP | GO:0044843 | cell cycle G1/S phase transition | 298/18862 | 0.002559541 | 0.007923606 | 6 |
| BP | GO:0009148 | pyrimidine nucleoside triphosphate biosynthetic process | 19/18862 | 0.003417474 | 0.010535071 | 2 |
| BP | GO:0006244 | pyrimidine nucleotide catabolic process | 20/18862 | 0.003785851 | 0.011621836 | 2 |
| BP | GO:0032869 | cellular response to insulin stimulus | 226/18862 | 0.00393724 | 0.012036212 | 5 |
| BP | GO:0071214 | cellular response to abiotic stimulus | 330/18862 | 0.00422173 | 0.012750831 | 6 |
| BP | GO:0104004 | cellular response to environmental stimulus | 330/18862 | 0.00422173 | 0.012750831 | 6 |
| BP | GO:0009581 | detection of external stimulus | 142/18862 | 0.004227989 | 0.012750831 | 4 |
| BP | GO:0007188 | adenylate cyclase-modulating G protein-coupled receptor signaling pathway | 230/18862 | 0.00424052 | 0.012750831 | 5 |
| BP | GO:0007189 | adenylate cyclase-activating G protein-coupled receptor signaling pathway | 143/18862 | 0.004334582 | 0.012980466 | 4 |
| BP | GO:0050891 | multicellular organismal water homeostasis | 72/18862 | 0.004494094 | 0.013403437 | 3 |
| BP | GO:0009582 | detection of abiotic stimulus | 145/18862 | 0.004553184 | 0.013524694 | 4 |
| BP | GO:0006359 | regulation of transcription by RNA polymerase III | 23/18862 | 0.004996147 | 0.014721261 | 2 |
| BP | GO:0006541 | glutamine metabolic process | 23/18862 | 0.004996147 | 0.014721261 | 2 |
| BP | GO:0006090 | pyruvate metabolic process | 150/18862 | 0.005131945 | 0.015060909 | 4 |
| BP | GO:0030104 | water homeostasis | 79/18862 | 0.005822794 | 0.01701049 | 3 |
| BP | GO:0034035 | purine ribonucleoside bisphosphate metabolic process | 25/18862 | 0.005888998 | 0.01701049 | 2 |
| BP | GO:0045745 | positive regulation of G protein-coupled receptor signaling pathway | 25/18862 | 0.005888998 | 0.01701049 | 2 |
| BP | GO:0050427 | 3'-phosphoadenosine 5'-phosphosulfate metabolic process | 25/18862 | 0.005888998 | 0.01701049 | 2 |
| BP | GO:0071880 | adenylate cyclase-activating adrenergic receptor signaling pathway | 26/18862 | 0.006360729 | 0.018301044 | 2 |
| BP | GO:0008380 | RNA splicing | 481/18862 | 0.006792955 | 0.019401883 | 7 |
| BP | GO:0006735 | NADH regeneration | 27/18862 | 0.006849115 | 0.019401883 | 2 |
| BP | GO:0061621 | canonical glycolysis | 27/18862 | 0.006849115 | 0.019401883 | 2 |
| BP | GO:0061718 | glucose catabolic process to pyruvate | 27/18862 | 0.006849115 | 0.019401883 | 2 |
| BP | GO:0043312 | neutrophil degranulation | 485/18862 | 0.00709594 | 0.020023765 | 7 |
| BP | GO:0002283 | neutrophil activation involved in immune response | 488/18862 | 0.007329704 | 0.020593556 | 7 |
| BP | GO:0061620 | glycolytic process through glucose-6-phosphate | 28/18862 | 0.007353997 | 0.020593556 | 2 |
| BP | GO:0061615 | glycolytic process through fructose-6-phosphate | 29/18862 | 0.007875216 | 0.021969282 | 2 |
| BP | GO:0002446 | neutrophil mediated immunity | 499/18862 | 0.008236087 | 0.022888965 | 7 |
| BP | GO:0042119 | neutrophil activation | 500/18862 | 0.008322414 | 0.023041598 | 7 |
| BP | GO:0005996 | monosaccharide metabolic process | 272/18862 | 0.00850188 | 0.023449981 | 5 |
| BP | GO:0071875 | adrenergic receptor signaling pathway | 31/18862 | 0.008966031 | 0.024637585 | 2 |
| BP | GO:0032868 | response to insulin | 278/18862 | 0.009288977 | 0.025429761 | 5 |
| BP | GO:0035590 | purinergic nucleotide receptor signaling pathway | 32/18862 | 0.009535315 | 0.026007101 | 2 |
| BP | GO:0006356 | regulation of transcription by RNA polymerase I | 33/18862 | 0.010120311 | 0.027500415 | 2 |
| BP | GO:0000083 | regulation of transcription involved in G1/S transition of mitotic cell cycle | 35/18862 | 0.011336823 | 0.030579588 | 2 |
| BP | GO:0000291 | nuclear-transcribed mRNA catabolic process, exonucleolytic | 35/18862 | 0.011336823 | 0.030579588 | 2 |
| BP | GO:0006007 | glucose catabolic process | 36/18862 | 0.011968035 | 0.03216395 | 2 |
| BP | GO:0071868 | cellular response to monoamine stimulus | 104/18862 | 0.012350051 | 0.032949226 | 3 |
| BP | GO:0071870 | cellular response to catecholamine stimulus | 104/18862 | 0.012350051 | 0.032949226 | 3 |
| BP | GO:0043949 | regulation of cAMP-mediated signaling | 37/18862 | 0.012614351 | 0.033532427 | 2 |
| BP | GO:0042417 | dopamine metabolic process | 38/18862 | 0.01327562 | 0.035162863 | 2 |
| BP | GO:0071867 | response to monoamine | 108/18862 | 0.013665106 | 0.035935026 | 3 |
| BP | GO:0071869 | response to catecholamine | 108/18862 | 0.013665106 | 0.035935026 | 3 |
| BP | GO:0006284 | base-excision repair | 39/18862 | 0.013951695 | 0.036427539 | 2 |
| BP | GO:0045742 | positive regulation of epidermal growth factor receptor signaling pathway | 39/18862 | 0.013951695 | 0.036427539 | 2 |
| BP | GO:0040029 | regulation of gene expression, epigenetic | 205/18862 | 0.014965612 | 0.038936289 | 4 |
| BP | GO:1901186 | positive regulation of ERBB signaling pathway | 41/18862 | 0.015347673 | 0.039789206 | 2 |
| BP | GO:0009416 | response to light stimulus | 319/18862 | 0.016050283 | 0.041362501 | 5 |
| BP | GO:0043114 | regulation of vascular permeability | 42/18862 | 0.016067284 | 0.041362501 | 2 |
| BP | GO:0006006 | glucose metabolic process | 210/18862 | 0.016210137 | 0.041584342 | 4 |
| BP | GO:0006734 | NADH metabolic process | 43/18862 | 0.016801118 | 0.042950226 | 2 |
| BP | GO:0032147 | activation of protein kinase activity | 328/18862 | 0.017882384 | 0.045555634 | 5 |
| BP | GO:0005978 | glycogen biosynthetic process | 46/18862 | 0.019086522 | 0.048121925 | 2 |
| BP | GO:0007595 | lactation | 46/18862 | 0.019086522 | 0.048121925 | 2 |
| BP | GO:0009250 | glucan biosynthetic process | 46/18862 | 0.019086522 | 0.048121925 | 2 |
| BP | GO:0045089 | positive regulation of innate immune response | 223/18862 | 0.019744572 | 0.049610549 | 4 |
| BP | GO:0033628 | regulation of cell adhesion mediated by integrin | 48/18862 | 0.020678635 | 0.051780163 | 2 |
| BP | GO:0045005 | DNA-dependent DNA replication maintenance of fidelity | 49/18862 | 0.021494826 | 0.053459031 | 2 |
| BP | GO:0046638 | positive regulation of alpha-beta T cell differentiation | 49/18862 | 0.021494826 | 0.053459031 | 2 |
| BP | GO:0045744 | negative regulation of G protein-coupled receptor signaling pathway | 50/18862 | 0.022324256 | 0.055334304 | 2 |
| BP | GO:0006584 | catecholamine metabolic process | 52/18862 | 0.02402229 | 0.059143539 | 2 |
| BP | GO:0009712 | catechol-containing compound metabolic process | 52/18862 | 0.02402229 | 0.059143539 | 2 |
| BP | GO:0031349 | positive regulation of defense response | 361/18862 | 0.025771108 | 0.063236974 | 5 |
| BP | GO:0016311 | dephosphorylation | 491/18862 | 0.025961257 | 0.063491214 | 6 |
| BP | GO:0008016 | regulation of heart contraction | 245/18862 | 0.026741218 | 0.065181427 | 4 |
| BP | GO:0006754 | ATP biosynthetic process | 56/18862 | 0.027571289 | 0.066982183 | 2 |
| BP | GO:2000242 | negative regulation of reproductive process | 57/18862 | 0.028489626 | 0.068816655 | 2 |
| BP | GO:0019318 | hexose metabolic process | 250/18862 | 0.028513988 | 0.068816655 | 4 |
| BP | GO:1903779 | regulation of cardiac conduction | 59/18862 | 0.030362694 | 0.073038127 | 2 |
| BP | GO:0002833 | positive regulation of response to biotic stimulus | 258/18862 | 0.031493456 | 0.075510624 | 4 |
| BP | GO:0032623 | interleukin-2 production | 61/18862 | 0.032283428 | 0.077152579 | 2 |
| BP | GO:0035567 | non-canonical Wnt signaling pathway | 153/18862 | 0.033824815 | 0.080573808 | 3 |
| BP | GO:0046635 | positive regulation of alpha-beta T cell activation | 66/18862 | 0.037287189 | 0.088534051 | 2 |
| BP | GO:0090501 | RNA phosphodiester bond hydrolysis | 160/18862 | 0.037847101 | 0.089573615 | 3 |
| BP | GO:0046637 | regulation of alpha-beta T cell differentiation | 67/18862 | 0.038321565 | 0.090404911 | 2 |
| BP | GO:0060047 | heart contraction | 279/18862 | 0.040160564 | 0.094439653 | 4 |
| BP | GO:0007584 | response to nutrient | 165/18862 | 0.04086361 | 0.095785896 | 3 |
| BP | GO:0033692 | cellular polysaccharide biosynthetic process | 71/18862 | 0.042567108 | 0.099461194 | 2 |
| BP | GO:0033627 | cell adhesion mediated by integrin | 72/18862 | 0.043654908 | 0.101679101 | 2 |
| BP | GO:0003015 | heart process | 289/18862 | 0.044721152 | 0.102653581 | 4 |
| BP | GO:0031100 | animal organ regeneration | 73/18862 | 0.04475304 | 0.102653581 | 2 |
| BP | GO:0007191 | adenylate cyclase-activating dopamine receptor signaling pathway | 10/18862 | 0.045189363 | 0.102653581 | 1 |
| BP | GO:0009176 | pyrimidine deoxyribonucleoside monophosphate metabolic process | 10/18862 | 0.045189363 | 0.102653581 | 1 |
| BP | GO:0009629 | response to gravity | 10/18862 | 0.045189363 | 0.102653581 | 1 |
| BP | GO:0043084 | penile erection | 10/18862 | 0.045189363 | 0.102653581 | 1 |
| BP | GO:0106072 | negative regulation of adenylate cyclase-activating G protein-coupled receptor signaling pathway | 10/18862 | 0.045189363 | 0.102653581 | 1 |
| BP | GO:1903522 | regulation of blood circulation | 290/18862 | 0.045192613 | 0.102653581 | 4 |
| BP | GO:0005977 | glycogen metabolic process | 74/18862 | 0.045861386 | 0.103851157 | 2 |
| BP | GO:0006073 | cellular glucan metabolic process | 75/18862 | 0.046979832 | 0.105407832 | 2 |
| BP | GO:0009064 | glutamine family amino acid metabolic process | 75/18862 | 0.046979832 | 0.105407832 | 2 |
| BP | GO:0044042 | glucan metabolic process | 75/18862 | 0.046979832 | 0.105407832 | 2 |
| BP | GO:0000288 | nuclear-transcribed mRNA catabolic process, deadenylation-dependent decay | 77/18862 | 0.049246567 | 0.108619677 | 2 |
| BP | GO:0001821 | histamine secretion | 11/18862 | 0.049595713 | 0.108619677 | 1 |
| BP | GO:0014870 | response to muscle inactivity | 11/18862 | 0.049595713 | 0.108619677 | 1 |
| BP | GO:0033197 | response to vitamin E | 11/18862 | 0.049595713 | 0.108619677 | 1 |
| BP | GO:0033632 | regulation of cell-cell adhesion mediated by integrin | 11/18862 | 0.049595713 | 0.108619677 | 1 |
| BP | GO:0045602 | negative regulation of endothelial cell differentiation | 11/18862 | 0.049595713 | 0.108619677 | 1 |
| BP | GO:0046710 | GDP metabolic process | 11/18862 | 0.049595713 | 0.108619677 | 1 |
| BP | GO:0070243 | regulation of thymocyte apoptotic process | 11/18862 | 0.049595713 | 0.108619677 | 1 |
| CC | GO:0061695 | transferase complex, transferring phosphorus-containing groups | 253/19520 | 7.92E-32 | 7.92E-30 | 28 |
| CC | GO:0055029 | nuclear DNA-directed RNA polymerase complex | 103/19520 | 4.82E-26 | 1.96E-24 | 19 |
| CC | GO:0000428 | DNA-directed RNA polymerase complex | 104/19520 | 5.88E-26 | 1.96E-24 | 19 |
| CC | GO:0030880 | RNA polymerase complex | 108/19520 | 1.27E-25 | 3.18E-24 | 19 |
| CC | GO:0005666 | RNA polymerase III complex | 18/19520 | 2.22E-22 | 4.44E-21 | 11 |
| CC | GO:0005665 | RNA polymerase II, core complex | 15/19520 | 7.02E-16 | 1.17E-14 | 8 |
| CC | GO:0042575 | DNA polymerase complex | 20/19520 | 1.35E-14 | 1.93E-13 | 8 |
| CC | GO:0005736 | RNA polymerase I complex | 13/19520 | 1.96E-09 | 2.45E-08 | 5 |
| CC | GO:0016591 | RNA polymerase II, holoenzyme | 79/19520 | 2.26E-09 | 2.51E-08 | 8 |
| CC | GO:0042622 | photoreceptor outer segment membrane | 16/19520 | 6.58E-09 | 6.58E-08 | 5 |
| CC | GO:0043601 | nuclear replisome | 22/19520 | 3.88E-08 | 3.53E-07 | 5 |
| CC | GO:0030894 | replisome | 24/19520 | 6.22E-08 | 5.19E-07 | 5 |
| CC | GO:0043596 | nuclear replication fork | 35/19520 | 4.57E-07 | 3.52E-06 | 5 |
| CC | GO:0097381 | photoreceptor disc membrane | 23/19520 | 3.06E-06 | 2.18E-05 | 4 |
| CC | GO:0005657 | replication fork | 65/19520 | 1.05E-05 | 6.99E-05 | 5 |
| CC | GO:0000228 | nuclear chromosome | 250/19520 | 1.58E-05 | 9.88E-05 | 8 |
| CC | GO:0060170 | ciliary membrane | 72/19520 | 1.73E-05 | 0.000101898 | 5 |
| CC | GO:0001750 | photoreceptor outer segment | 89/19520 | 4.84E-05 | 0.000269043 | 5 |
| CC | GO:0097733 | photoreceptor cell cilium | 115/19520 | 0.000163543 | 0.000860755 | 5 |
| CC | GO:0101002 | ficolin-1-rich granule | 185/19520 | 0.000178624 | 0.000893122 | 6 |
| CC | GO:1904813 | ficolin-1-rich granule lumen | 124/19520 | 0.000232515 | 0.001107213 | 5 |
| CC | GO:0097731 | 9+0 non-motile cilium | 126/19520 | 0.000250461 | 0.001138459 | 5 |
| CC | GO:0097730 | non-motile cilium | 160/19520 | 0.000747524 | 0.003250104 | 5 |
| CC | GO:0005671 | Ada2/Gcn5/Ada3 transcription activator complex | 15/19520 | 0.001985576 | 0.008273232 | 2 |
| CC | GO:0032993 | protein-DNA complex | 208/19520 | 0.002384722 | 0.00953889 | 5 |
| CC | GO:0060205 | cytoplasmic vesicle lumen | 326/19520 | 0.003369074 | 0.012857364 | 6 |
| CC | GO:0031983 | vesicle lumen | 328/19520 | 0.003471488 | 0.012857364 | 6 |
| CC | GO:0034774 | secretory granule lumen | 322/19520 | 0.014565934 | 0.052021192 | 5 |
| CC | GO:0031253 | cell projection membrane | 337/19520 | 0.017398021 | 0.059993176 | 5 |
| CC | GO:1902562 | H4 histone acetyltransferase complex | 47/19520 | 0.018642152 | 0.062140505 | 2 |
| CC | GO:0031010 | ISWI-type complex | 11/19520 | 0.047960581 | 0.154711553 | 1 |
| MF | GO:0016779 | nucleotidyltransferase activity | 131/18337 | 5.52E-32 | 3.66E-30 | 24 |
| MF | GO:0003899 | DNA-directed 5'-3' RNA polymerase activity | 39/18337 | 6.46E-28 | 2.14E-26 | 16 |
| MF | GO:0034062 | 5'-3' RNA polymerase activity | 43/18337 | 4.48E-27 | 7.42E-26 | 16 |
| MF | GO:0097747 | RNA polymerase activity | 43/18337 | 4.48E-27 | 7.42E-26 | 16 |
| MF | GO:0004114 | 3',5'-cyclic-nucleotide phosphodiesterase activity | 23/18337 | 4.16E-18 | 5.51E-17 | 10 |
| MF | GO:0004112 | cyclic-nucleotide phosphodiesterase activity | 25/18337 | 1.18E-17 | 1.30E-16 | 10 |
| MF | GO:0009975 | cyclase activity | 22/18337 | 6.15E-14 | 5.10E-13 | 8 |
| MF | GO:0016849 | phosphorus-oxygen lyase activity | 22/18337 | 6.15E-14 | 5.10E-13 | 8 |
| MF | GO:0047555 | 3',5'-cyclic-GMP phosphodiesterase activity | 14/18337 | 1.53E-13 | 1.13E-12 | 7 |
| MF | GO:0140098 | catalytic activity, acting on RNA | 386/18337 | 2.80E-13 | 1.86E-12 | 18 |
| MF | GO:0008081 | phosphoric diester hydrolase activity | 89/18337 | 4.39E-13 | 2.65E-12 | 11 |
| MF | GO:0042578 | phosphoric ester hydrolase activity | 367/18337 | 1.54E-12 | 8.54E-12 | 17 |
| MF | GO:0017110 | nucleoside-diphosphatase activity | 14/18337 | 2.99E-11 | 1.53E-10 | 6 |
| MF | GO:0000287 | magnesium ion binding | 216/18337 | 3.23E-11 | 1.53E-10 | 13 |
| MF | GO:0019205 | nucleobase-containing compound kinase activity | 42/18337 | 1.08E-09 | 4.79E-09 | 7 |
| MF | GO:0003887 | DNA-directed DNA polymerase activity | 25/18337 | 1.69E-09 | 7.01E-09 | 6 |
| MF | GO:0008253 | 5'-nucleotidase activity | 13/18337 | 2.83E-09 | 1.11E-08 | 5 |
| MF | GO:0008252 | nucleotidase activity | 15/18337 | 6.56E-09 | 2.42E-08 | 5 |
| MF | GO:0034061 | DNA polymerase activity | 36/18337 | 1.78E-08 | 6.22E-08 | 6 |
| MF | GO:0016776 | phosphotransferase activity, phosphate group as acceptor | 39/18337 | 2.95E-08 | 9.79E-08 | 6 |
| MF | GO:0004115 | 3',5'-cyclic-AMP phosphodiesterase activity | 13/18337 | 3.43E-07 | 1.08E-06 | 4 |
| MF | GO:0019001 | guanyl nucleotide binding | 398/18337 | 4.30E-07 | 1.24E-06 | 12 |
| MF | GO:0032561 | guanyl ribonucleotide binding | 398/18337 | 4.30E-07 | 1.24E-06 | 12 |
| MF | GO:0030553 | cGMP binding | 15/18337 | 6.49E-07 | 1.79E-06 | 4 |
| MF | GO:0030551 | cyclic nucleotide binding | 37/18337 | 8.76E-07 | 2.32E-06 | 5 |
| MF | GO:0004550 | nucleoside diphosphate kinase activity | 18/18337 | 1.44E-06 | 3.67E-06 | 4 |
| MF | GO:0001882 | nucleoside binding | 390/18337 | 2.58E-06 | 6.34E-06 | 11 |
| MF | GO:0016829 | lyase activity | 194/18337 | 4.24E-06 | 1.00E-05 | 8 |
| MF | GO:0004016 | adenylate cyclase activity | 10/18337 | 1.25E-05 | 2.86E-05 | 3 |
| MF | GO:0019239 | deaminase activity | 32/18337 | 1.61E-05 | 3.55E-05 | 4 |
| MF | GO:0047429 | nucleoside-triphosphate diphosphatase activity | 11/18337 | 1.71E-05 | 3.67E-05 | 3 |
| MF | GO:0016814 | hydrolase activity, acting on carbon-nitrogen (but not peptide) bonds, in cyclic amidines | 35/18337 | 2.31E-05 | 4.80E-05 | 4 |
| MF | GO:0001883 | purine nucleoside binding | 384/18337 | 9.38E-05 | 0.000182866 | 9 |
| MF | GO:0032549 | ribonucleoside binding | 384/18337 | 9.38E-05 | 0.000182866 | 9 |
| MF | GO:0004551 | nucleotide diphosphatase activity | 20/18337 | 0.000114766 | 0.000211411 | 3 |
| MF | GO:0050145 | nucleoside monophosphate kinase activity | 20/18337 | 0.000114766 | 0.000211411 | 3 |
| MF | GO:0016810 | hydrolase activity, acting on carbon-nitrogen (but not peptide) bonds | 117/18337 | 0.000249308 | 0.00044684 | 5 |
| MF | GO:0051536 | iron-sulfur cluster binding | 67/18337 | 0.000301384 | 0.000512475 | 4 |
| MF | GO:0051540 | metal cluster binding | 67/18337 | 0.000301384 | 0.000512475 | 4 |
| MF | GO:0140097 | catalytic activity, acting on DNA | 204/18337 | 0.000447472 | 0.000741861 | 6 |
| MF | GO:0032550 | purine ribonucleoside binding | 381/18337 | 0.000487782 | 0.000788967 | 8 |
| MF | GO:0042562 | hormone binding | 84/18337 | 0.000713134 | 0.001126001 | 4 |
| MF | GO:0016884 | carbon-nitrogen ligase activity, with glutamine as amido-N-donor | 10/18337 | 0.000999341 | 0.001541211 | 2 |
| MF | GO:0051539 | 4 iron, 4 sulfur cluster binding | 42/18337 | 0.001070896 | 0.001614029 | 3 |
| MF | GO:0008296 | 3'-5'-exodeoxyribonuclease activity | 11/18337 | 0.001217608 | 0.001755362 | 2 |
| MF | GO:0030955 | potassium ion binding | 11/18337 | 0.001217608 | 0.001755362 | 2 |
| MF | GO:0008179 | adenylate cyclase binding | 12/18337 | 0.001456575 | 0.002012374 | 2 |
| MF | GO:0042301 | phosphate ion binding | 12/18337 | 0.001456575 | 0.002012374 | 2 |
| MF | GO:0016763 | transferase activity, transferring pentosyl groups | 50/18337 | 0.001778522 | 0.002358882 | 3 |
| MF | GO:0017046 | peptide hormone binding | 50/18337 | 0.001778522 | 0.002358882 | 3 |
| MF | GO:0016791 | phosphatase activity | 276/18337 | 0.002127892 | 0.002766918 | 6 |
| MF | GO:0005525 | GTP binding | 376/18337 | 0.002204154 | 0.002810965 | 7 |
| MF | GO:0016208 | AMP binding | 15/18337 | 0.002295692 | 0.002872465 | 2 |
| MF | GO:0031420 | alkali metal ion binding | 16/18337 | 0.002615482 | 0.003211995 | 2 |
| MF | GO:0004529 | exodeoxyribonuclease activity | 22/18337 | 0.004941618 | 0.005851916 | 2 |
| MF | GO:0016895 | exodeoxyribonuclease activity, producing 5'-phosphomonoesters | 22/18337 | 0.004941618 | 0.005851916 | 2 |
| MF | GO:0030552 | cAMP binding | 23/18337 | 0.005395445 | 0.006277248 | 2 |
| MF | GO:0001653 | peptide receptor activity | 152/18337 | 0.006174559 | 0.00705984 | 4 |
| MF | GO:0004527 | exonuclease activity | 82/18337 | 0.007198266 | 0.008090825 | 3 |
| MF | GO:0030515 | snoRNA binding | 32/18337 | 0.010286219 | 0.011368979 | 2 |
| MF | GO:0043531 | ADP binding | 39/18337 | 0.015037765 | 0.016348218 | 2 |
| MF | GO:0004518 | nuclease activity | 206/18337 | 0.017336718 | 0.018543518 | 4 |
| MF | GO:0003697 | single-stranded DNA binding | 116/18337 | 0.018342166 | 0.019307543 | 3 |
| MF | GO:0016879 | ligase activity, forming carbon-nitrogen bonds | 47/18337 | 0.021402638 | 0.022177075 | 2 |
| MF | GO:0008408 | 3'-5' exonuclease activity | 55/18337 | 0.028686422 | 0.029267119 | 2 |
| MF | GO:0016796 | exonuclease activity, active with either ribo- or deoxyribonucleic acids and producing 5'-phosphomonoesters | 57/18337 | 0.030641844 | 0.030788456 | 2 |
| MF | GO:0004536 | deoxyribonuclease activity | 59/18337 | 0.032648716 | 0.032315304 | 2 |
| MF | GO:0030145 | manganese ion binding | 62/18337 | 0.035753003 | 0.034867479 | 2 |
| MF | GO:0015643 | toxic substance binding | 10/18337 | 0.046978451 | 0.044505901 | 1 |
| MF | GO:0016868 | intramolecular transferase activity, phosphotransferases | 10/18337 | 0.046978451 | 0.044505901 | 1 |

**Table S3b. Analysis of KEGG.**

| ID | Description | GeneRatio | BgRatio | pvalue | qvalue | Count |
| --- | --- | --- | --- | --- | --- | --- |
| hsa00230 | Purine metabolism | 82/83 | 162/5894 | 1.66E-137 | 5.42E-136 | 82 |
| hsa00240 | Pyrimidine metabolism | 39/83 | 99/5894 | 5.34E-50 | 8.72E-49 | 39 |
| hsa03020 | RNA polymerase | 17/83 | 29/5894 | 2.71E-25 | 2.95E-24 | 17 |
| hsa03030 | DNA replication | 8/83 | 36/5894 | 2.41E-08 | 1.97E-07 | 8 |
| hsa00760 | Nicotinate and nicotinamide metabolism | 6/83 | 24/5894 | 7.14E-07 | 4.66E-06 | 6 |
| hsa04623 | Cytosolic DNA-sensing pathway | 8/83 | 56/5894 | 9.02E-07 | 4.91E-06 | 8 |
| hsa03410 | Base excision repair | 6/83 | 34/5894 | 6.38E-06 | 2.97E-05 | 6 |
| hsa03420 | Nucleotide excision repair | 6/83 | 45/5894 | 3.41E-05 | 0.000139219 | 6 |
| hsa04744 | Phototransduction | 4/83 | 29/5894 | 0.000664163 | 0.002408077 | 4 |
| hsa00983 | Drug metabolism - other enzymes | 5/83 | 52/5894 | 0.000758603 | 0.002475442 | 5 |
| hsa03430 | Mismatch repair | 3/83 | 23/5894 | 0.003891744 | 0.01088077 | 3 |
| hsa05016 | Huntington's disease | 8/83 | 183/5894 | 0.004001316 | 0.01088077 | 8 |
| hsa04270 | Vascular smooth muscle contraction | 6/83 | 116/5894 | 0.005623275 | 0.014115104 | 6 |
| hsa00030 | Pentose phosphate pathway | 3/83 | 27/5894 | 0.006172871 | 0.014387894 | 3 |
| hsa03440 | Homologous recombination | 3/83 | 28/5894 | 0.006844169 | 0.01488907 | 3 |
| hsa04914 | Progesterone-mediated oocyte maturation | 4/83 | 87/5894 | 0.033688976 | 0.067944588 | 4 |
| hsa04970 | Salivary secretion | 4/83 | 89/5894 | 0.036189469 | 0.067944588 | 4 |
| hsa04540 | Gap junction | 4/83 | 90/5894 | 0.037479111 | 0.067944588 | 4 |

# Appendix 4

**LASSO and** **SVM-RFE genes**

**Table S4a. LASSO genes.**

| PGM1 | POLR2K | PDE6H | ENTPD1 |
| --- | --- | --- | --- |
| PRPS2 | POLR3B | AK3 | PDE6D |
| NT5C1A | NPR2 | PDE4B | ALLC |
| PNP |  |  |  |

**Table S4b. SVM-RFE genes.**

| NPR2 | NUDT16 | PDE6H | PDE6D |
| --- | --- | --- | --- |
| ENTPD1 | PDE4B | GUCY1B3 | POLR2J |
| PDE7A | ALLC | POLR2K |  |

**Table S4c. InterGenes.**

| ENTPD1 | PDE6D | NPR2 | ALLC |
| --- | --- | --- | --- |
| POLR2K | PDE6H | PDE4B |  |

# Appendix 5

**GSEA analysis**

**Table 5a. PDE4B of GSEA analysis.**

| ID | setSize | NES | pvalue | qvalues | rank |
| --- | --- | --- | --- | --- | --- |
| KEGG_NEUROACTIVE_LIGAND_RECEPTOR_INTERACTION | 262 | -2.464222215 | 1.00E-10 | 3.40E-09 | 6320 |
| KEGG_SPLICEOSOME | 115 | 2.697414531 | 1.00E-10 | 3.40E-09 | 6442 |
| KEGG_UBIQUITIN_MEDIATED_PROTEOLYSIS | 125 | 2.570098549 | 1.00E-10 | 3.40E-09 | 5394 |
| KEGG_PROTEIN_EXPORT | 22 | 2.613606385 | 2.50E-09 | 6.37E-08 | 3930 |
| KEGG_OLFACTORY_TRANSDUCTION | 108 | -2.209254872 | 2.45E-07 | 4.99E-06 | 7723 |
| KEGG_RNA_DEGRADATION | 54 | 2.351203184 | 5.63E-07 | 9.58E-06 | 5513 |
| KEGG_T_CELL_RECEPTOR_SIGNALING_PATHWAY | 107 | 2.140172381 | 1.29E-06 | 1.88E-05 | 5816 |
| KEGG_PROTEASOME | 40 | 2.33920674 | 2.11E-06 | 2.69E-05 | 4838 |
| KEGG_PPAR_SIGNALING_PATHWAY | 66 | -2.179396121 | 4.59E-06 | 5.21E-05 | 3065 |
| KEGG_LYSOSOME | 116 | 2.022717177 | 9.11E-06 | 9.30E-05 | 6295 |
| KEGG_PANCREATIC_CANCER | 69 | 2.069142633 | 1.61E-05 | 0.000138838 | 5277 |
| KEGG_N_GLYCAN_BIOSYNTHESIS | 44 | 2.143511628 | 1.63E-05 | 0.000138838 | 5117 |
| KEGG_B_CELL_RECEPTOR_SIGNALING_PATHWAY | 75 | 2.060144418 | 2.11E-05 | 0.000165849 | 5887 |
| KEGG_ENDOMETRIAL_CANCER | 52 | 2.107146955 | 3.68E-05 | 0.000268165 | 5816 |
| KEGG_BASAL_TRANSCRIPTION_FACTORS | 32 | 2.12219906 | 4.45E-05 | 0.000302998 | 4194 |
| KEGG_RIBOSOME | 69 | 2.009693071 | 4.95E-05 | 0.000316199 | 8043 |
| KEGG_COLORECTAL_CANCER | 62 | 2.013862554 | 6.04E-05 | 0.000362975 | 5277 |
| KEGG_OXIDATIVE_PHOSPHORYLATION | 95 | 1.967862409 | 6.86E-05 | 0.000389262 | 6617 |
| KEGG_CALCIUM_SIGNALING_PATHWAY | 172 | -1.752095092 | 9.42E-05 | 0.000506172 | 5837 |
| KEGG_CELL_CYCLE | 122 | 1.900886024 | 0.000114179 | 0.000582915 | 7230 |
| KEGG_PRIMARY_IMMUNODEFICIENCY | 35 | 2.090515472 | 0.000123913 | 0.000602486 | 2648 |
| KEGG_PYRIMIDINE_METABOLISM | 91 | 1.864474409 | 0.000182924 | 0.000848977 | 4381 |
| KEGG_VIBRIO_CHOLERAE_INFECTION | 51 | 1.948445456 | 0.000297327 | 0.00131994 | 6471 |
| KEGG_NOD_LIKE_RECEPTOR_SIGNALING_PATHWAY | 60 | 1.894497527 | 0.000330831 | 0.001401765 | 5789 |
| KEGG_RETINOL_METABOLISM | 45 | -1.976160652 | 0.000343216 | 0.001401765 | 6610 |
| KEGG_COMPLEMENT_AND_COAGULATION_CASCADES | 67 | -1.88570234 | 0.000381896 | 0.001499755 | 6849 |
| KEGG_DILATED_CARDIOMYOPATHY | 88 | -1.7936465 | 0.000413681 | 0.001564408 | 5200 |
| KEGG_HUNTINGTONS_DISEASE | 152 | 1.765955443 | 0.000445556 | 0.001606047 | 6327 |
| KEGG_PATHOGENIC_ESCHERICHIA_COLI_INFECTION | 51 | 1.910435475 | 0.00045615 | 0.001606047 | 5878 |
| KEGG_APOPTOSIS | 87 | 1.842643216 | 0.000491796 | 0.001673833 | 4893 |
| KEGG_ADHERENS_JUNCTION | 66 | 1.888216485 | 0.000548824 | 0.001807672 | 5876 |
| KEGG_PARKINSONS_DISEASE | 93 | 1.808066573 | 0.00058506 | 0.001866802 | 6668 |
| KEGG_NON_SMALL_CELL_LUNG_CANCER | 54 | 1.886808792 | 0.00062685 | 0.001939535 | 5816 |
| KEGG_EPITHELIAL_CELL_SIGNALING_IN_HELICOBACTER_PYLORI_INFECTION | 67 | 1.78692489 | 0.001056316 | 0.003172219 | 6499 |
| KEGG_INTESTINAL_IMMUNE_NETWORK_FOR_IGA_PRODUCTION | 44 | 1.829997787 | 0.001277747 | 0.003727563 | 2201 |
| KEGG_CHRONIC_MYELOID_LEUKEMIA | 72 | 1.751424109 | 0.001688662 | 0.004789479 | 5816 |
| KEGG_HYPERTROPHIC_CARDIOMYOPATHY_HCM | 81 | -1.693496697 | 0.001800871 | 0.004969687 | 6183 |
| KEGG_ACUTE_MYELOID_LEUKEMIA | 56 | 1.735750103 | 0.002257749 | 0.006066528 | 5816 |
| KEGG_PURINE_METABOLISM | 145 | 1.621582293 | 0.002558543 | 0.006646422 | 4381 |
| KEGG_LEISHMANIA_INFECTION | 67 | 1.700867423 | 0.002603753 | 0.006646422 | 6460 |
| KEGG_ENDOCYTOSIS | 170 | 1.565746738 | 0.003066158 | 0.007560812 | 5050 |
| KEGG_CYTOSOLIC_DNA_SENSING_PATHWAY | 50 | 1.738338375 | 0.003110066 | 0.007560812 | 4929 |
| KEGG_PROSTATE_CANCER | 89 | 1.698969555 | 0.003335806 | 0.007921008 | 5816 |
| KEGG_TOLL_LIKE_RECEPTOR_SIGNALING_PATHWAY | 97 | 1.653483623 | 0.00406484 | 0.009432762 | 3891 |
| KEGG_ALZHEIMERS_DISEASE | 138 | 1.607966141 | 0.004687018 | 0.010634872 | 6345 |
| KEGG_RENAL_CELL_CARCINOMA | 66 | 1.656943682 | 0.005845533 | 0.012454768 | 5816 |
| KEGG_P53_SIGNALING_PATHWAY | 65 | 1.648296151 | 0.005862428 | 0.012454768 | 4931 |
| KEGG_NEUROTROPHIN_SIGNALING_PATHWAY | 122 | 1.619482196 | 0.005865648 | 0.012454768 | 6120 |
| KEGG_RENIN_ANGIOTENSIN_SYSTEM | 17 | -1.830177196 | 0.006059542 | 0.012454768 | 6752 |
| KEGG_CHEMOKINE_SIGNALING_PATHWAY | 179 | 1.532269247 | 0.006098984 | 0.012454768 | 5542 |
| KEGG_GLYCOSYLPHOSPHATIDYLINOSITOL_GPI_ANCHOR_BIOSYNTHESIS | 24 | 1.77098533 | 0.00666695 | 0.013347661 | 3652 |
| KEGG_SPHINGOLIPID_METABOLISM | 31 | 1.685001462 | 0.00695494 | 0.013656462 | 2838 |
| KEGG_WNT_SIGNALING_PATHWAY | 146 | 1.568651662 | 0.007218373 | 0.0139063 | 4459 |
| KEGG_NUCLEOTIDE_EXCISION_REPAIR | 43 | 1.682009654 | 0.009722025 | 0.018182698 | 6596 |
| KEGG_MATURITY_ONSET_DIABETES_OF_THE_YOUNG | 23 | -1.734898397 | 0.009794288 | 0.018182698 | 4209 |
| KEGG_THYROID_CANCER | 29 | 1.690431414 | 0.010331222 | 0.018837002 | 3228 |
| KEGG_SNARE_INTERACTIONS_IN_VESICULAR_TRANSPORT | 37 | 1.702245941 | 0.010573981 | 0.018941388 | 7917 |
| KEGG_PHOSPHATIDYLINOSITOL_SIGNALING_SYSTEM | 74 | 1.548777254 | 0.01168147 | 0.020564475 | 4773 |
| KEGG_OOCYTE_MEIOSIS | 107 | 1.550493222 | 0.012110136 | 0.020957773 | 5443 |
| KEGG_MTOR_SIGNALING_PATHWAY | 50 | 1.577640322 | 0.012793945 | 0.021772152 | 5293 |
| KEGG_METABOLISM_OF_XENOBIOTICS_BY_CYTOCHROME_P450 | 55 | -1.585421237 | 0.015861749 | 0.026550296 | 5863 |
| KEGG_VASOPRESSIN_REGULATED_WATER_REABSORPTION | 44 | 1.617871079 | 0.016138792 | 0.026578316 | 3360 |
| KEGG_AMINOACYL_TRNA_BIOSYNTHESIS | 21 | 1.700158527 | 0.016667563 | 0.027013428 | 5463 |
| KEGG_TYROSINE_METABOLISM | 40 | -1.58150524 | 0.018506761 | 0.029525588 | 2902 |
| KEGG_HEDGEHOG_SIGNALING_PATHWAY | 53 | -1.498044134 | 0.020986804 | 0.032967125 | 6674 |
| KEGG_ALLOGRAFT_REJECTION | 33 | 1.594826295 | 0.021599089 | 0.033414858 | 7955 |
| KEGG_ERBB_SIGNALING_PATHWAY | 86 | 1.511758917 | 0.02228173 | 0.033933113 | 5816 |
| KEGG_AMINO_SUGAR_AND_NUCLEOTIDE_SUGAR_METABOLISM | 40 | 1.587485928 | 0.022863155 | 0.033933113 | 4502 |
| KEGG_ADIPOCYTOKINE_SIGNALING_PATHWAY | 65 | -1.468411039 | 0.022931088 | 0.033933113 | 1456 |
| KEGG_DNA_REPLICATION | 36 | 1.591650719 | 0.023768224 | 0.034299284 | 6596 |
| KEGG_ANTIGEN_PROCESSING_AND_PRESENTATION | 74 | 1.49766292 | 0.023909092 | 0.034299284 | 5631 |
| KEGG_HEMATOPOIETIC_CELL_LINEAGE | 83 | 1.531321147 | 0.024186299 | 0.034299284 | 2072 |
| KEGG_RIBOFLAVIN_METABOLISM | 14 | 1.698659528 | 0.025341069 | 0.03544461 | 4461 |
| KEGG_STEROID_BIOSYNTHESIS | 16 | 1.651301752 | 0.025862931 | 0.035685694 | 2762 |
| KEGG_O_GLYCAN_BIOSYNTHESIS | 28 | 1.561388435 | 0.028013436 | 0.03813759 | 2940 |
| KEGG_PEROXISOME | 77 | 1.455532819 | 0.029679365 | 0.03987394 | 6193 |
| KEGG_CELL_ADHESION_MOLECULES_CAMS | 125 | 1.432184123 | 0.034770515 | 0.045199334 | 5396 |
| KEGG_LEUKOCYTE_TRANSENDOTHELIAL_MIGRATION | 112 | 1.430383321 | 0.035211268 | 0.045199334 | 4700 |
| KEGG_BLADDER_CANCER | 41 | 1.511408805 | 0.035389585 | 0.045199334 | 5277 |
| KEGG_SMALL_CELL_LUNG_CANCER | 84 | 1.482016278 | 0.035413911 | 0.045199334 | 5996 |
| KEGG_CARDIAC_MUSCLE_CONTRACTION | 70 | -1.362211595 | 0.03593516 | 0.045298383 | 5554 |
| KEGG_FC_GAMMA_R_MEDIATED_PHAGOCYTOSIS | 90 | 1.416737307 | 0.036775106 | 0.045791852 | 6062 |
| KEGG_RIG_I_LIKE_RECEPTOR_SIGNALING_PATHWAY | 67 | 1.44237548 | 0.037681159 | 0.045860455 | 3889 |
| KEGG_CIRCADIAN_RHYTHM_MAMMAL | 11 | 1.604844503 | 0.037728498 | 0.045860455 | 4934 |
| KEGG_PATHWAYS_IN_CANCER | 318 | 1.292530212 | 0.039332539 | 0.047247756 | 5418 |
| KEGG_GLYCEROLIPID_METABOLISM | 42 | -1.446072072 | 0.046958774 | 0.055752768 | 5519 |
| KEGG_PROGESTERONE_MEDIATED_OOCYTE_MATURATION | 83 | 1.385615803 | 0.049275362 | 0.057830734 | 5443 |

**Table 5b. PDE6H of GSEA analysis.**

| ID | setSize | NES | pvalue | qvalues | rank |
| --- | --- | --- | --- | --- | --- |
| KEGG_NEUROACTIVE_LIGAND_RECEPTOR_INTERACTION | 262 | 2.114786871 | 1.00E-10 | 3.82E-09 | 5776 |
| KEGG_SPLICEOSOME | 115 | -2.657425185 | 1.00E-10 | 3.82E-09 | 7674 |
| KEGG_UBIQUITIN_MEDIATED_PROTEOLYSIS | 125 | -2.529274124 | 1.00E-10 | 3.82E-09 | 4087 |
| KEGG_B_CELL_RECEPTOR_SIGNALING_PATHWAY | 75 | -2.476325022 | 3.33E-10 | 9.55E-09 | 4973 |
| KEGG_CELL_CYCLE | 122 | -2.352124319 | 1.08E-09 | 2.48E-08 | 4608 |
| KEGG_T_CELL_RECEPTOR_SIGNALING_PATHWAY | 107 | -2.333270969 | 6.01E-09 | 1.15E-07 | 5425 |
| KEGG_OLFACTORY_TRANSDUCTION | 108 | 2.333604541 | 1.02E-08 | 1.67E-07 | 8360 |
| KEGG_PRIMARY_IMMUNODEFICIENCY | 35 | -2.477781733 | 2.70E-08 | 3.68E-07 | 2800 |
| KEGG_VIRAL_MYOCARDITIS | 65 | -2.359648968 | 2.89E-08 | 3.68E-07 | 5508 |
| KEGG_RNA_DEGRADATION | 54 | -2.330017941 | 3.36E-07 | 3.86E-06 | 5724 |
| KEGG_LEISHMANIA_INFECTION | 67 | -2.2447798 | 4.05E-07 | 4.22E-06 | 3901 |
| KEGG_HEMATOPOIETIC_CELL_LINEAGE | 83 | -2.124103003 | 1.26E-06 | 1.20E-05 | 2807 |
| KEGG_RIBOSOME | 69 | -2.186641839 | 1.52E-06 | 1.34E-05 | 7899 |
| KEGG_ALLOGRAFT_REJECTION | 33 | -2.289564816 | 1.93E-06 | 1.58E-05 | 5508 |
| KEGG_CHEMOKINE_SIGNALING_PATHWAY | 179 | -1.955031321 | 2.20E-06 | 1.68E-05 | 6093 |
| KEGG_APOPTOSIS | 87 | -2.08254712 | 3.85E-06 | 2.76E-05 | 4939 |
| KEGG_COLORECTAL_CANCER | 62 | -2.098575163 | 1.88E-05 | 0.000126697 | 5930 |
| KEGG_DNA_REPLICATION | 36 | -2.102275913 | 3.20E-05 | 0.000204133 | 5269 |
| KEGG_NATURAL_KILLER_CELL_MEDIATED_CYTOTOXICITY | 128 | -1.863122205 | 8.07E-05 | 0.000487307 | 5992 |
| KEGG_GRAFT_VERSUS_HOST_DISEASE | 35 | -2.060658779 | 0.000121408 | 0.000686078 | 5508 |
| KEGG_FC_GAMMA_R_MEDIATED_PHAGOCYTOSIS | 90 | -1.881465616 | 0.000125571 | 0.000686078 | 5823 |
| KEGG_LEUKOCYTE_TRANSENDOTHELIAL_MIGRATION | 112 | -1.829921336 | 0.000145969 | 0.000761272 | 4663 |
| KEGG_TYPE_I_DIABETES_MELLITUS | 39 | -1.994596126 | 0.0001643 | 0.000819622 | 5508 |
| KEGG_INTESTINAL_IMMUNE_NETWORK_FOR_IGA_PRODUCTION | 44 | -1.996700919 | 0.000174092 | 0.000820612 | 3991 |
| KEGG_NEUROTROPHIN_SIGNALING_PATHWAY | 122 | -1.79575872 | 0.000178803 | 0.000820612 | 6394 |
| KEGG_TOLL_LIKE_RECEPTOR_SIGNALING_PATHWAY | 97 | -1.816699599 | 0.000315112 | 0.001362798 | 6296 |
| KEGG_P53_SIGNALING_PATHWAY | 65 | -1.87968021 | 0.000320695 | 0.001362798 | 5002 |
| KEGG_PROTEASOME | 40 | -1.962891977 | 0.000359277 | 0.001472227 | 6902 |
| KEGG_PATHOGENIC_ESCHERICHIA_COLI_INFECTION | 51 | -1.905010734 | 0.000485351 | 0.001920263 | 4361 |
| KEGG_RENAL_CELL_CARCINOMA | 66 | -1.83187422 | 0.000539086 | 0.002061767 | 7163 |
| KEGG_NOD_LIKE_RECEPTOR_SIGNALING_PATHWAY | 60 | -1.864793977 | 0.000740852 | 0.002713581 | 6291 |
| KEGG_CHRONIC_MYELOID_LEUKEMIA | 72 | -1.846823927 | 0.000770366 | 0.002713581 | 6554 |
| KEGG_PROTEIN_EXPORT | 22 | -1.977940376 | 0.000780466 | 0.002713581 | 7611 |
| KEGG_PHOSPHATIDYLINOSITOL_SIGNALING_SYSTEM | 74 | -1.770931415 | 0.000827254 | 0.002791661 | 4971 |
| KEGG_MATURITY_ONSET_DIABETES_OF_THE_YOUNG | 23 | 2.018552688 | 0.000896568 | 0.002939124 | 4880 |
| KEGG_SYSTEMIC_LUPUS_ERYTHEMATOSUS | 50 | -1.86168675 | 0.00100568 | 0.003205237 | 4621 |
| KEGG_PANCREATIC_CANCER | 69 | -1.79982139 | 0.001157203 | 0.00358848 | 5930 |
| KEGG_LYSOSOME | 116 | -1.602753876 | 0.001535564 | 0.004636468 | 5751 |
| KEGG_ACUTE_MYELOID_LEUKEMIA | 56 | -1.793882874 | 0.001782722 | 0.005244715 | 6633 |
| KEGG_STEROID_HORMONE_BIOSYNTHESIS | 41 | 1.819578413 | 0.00184874 | 0.005302964 | 4407 |
| KEGG_NUCLEOTIDE_EXCISION_REPAIR | 43 | -1.80929723 | 0.0022483 | 0.006291777 | 6387 |
| KEGG_PYRIMIDINE_METABOLISM | 91 | -1.692821704 | 0.002388064 | 0.006330186 | 3968 |
| KEGG_ANTIGEN_PROCESSING_AND_PRESENTATION | 74 | -1.68869697 | 0.002394848 | 0.006330186 | 5508 |
| KEGG_JAK_STAT_SIGNALING_PATHWAY | 152 | -1.562559659 | 0.002427539 | 0.006330186 | 2996 |
| KEGG_AUTOIMMUNE_THYROID_DISEASE | 46 | -1.730929702 | 0.003107904 | 0.007924247 | 5508 |
| KEGG_N_GLYCAN_BIOSYNTHESIS | 44 | -1.775277129 | 0.003501648 | 0.008734087 | 3194 |
| KEGG_SMALL_CELL_LUNG_CANCER | 84 | -1.653665479 | 0.004021511 | 0.009817351 | 5034 |
| KEGG_INOSITOL_PHOSPHATE_METABOLISM | 54 | -1.706945918 | 0.004215652 | 0.010076887 | 5142 |
| KEGG_BASE_EXCISION_REPAIR | 32 | -1.768872204 | 0.004621213 | 0.010820885 | 3904 |
| KEGG_FC_EPSILON_RI_SIGNALING_PATHWAY | 73 | -1.6893617 | 0.007197585 | 0.016516563 | 5882 |
| KEGG_HEDGEHOG_SIGNALING_PATHWAY | 53 | 1.633389091 | 0.0075795 | 0.017051921 | 6818 |
| KEGG_MISMATCH_REPAIR | 22 | -1.78450071 | 0.007984766 | 0.017331504 | 5269 |
| KEGG_PARKINSONS_DISEASE | 93 | -1.558355352 | 0.008005883 | 0.017331504 | 7062 |
| KEGG_ENDOCYTOSIS | 170 | -1.477846056 | 0.01054603 | 0.022407744 | 5600 |
| KEGG_NON_HOMOLOGOUS_END_JOINING | 11 | -1.728902036 | 0.018079922 | 0.037003608 | 3153 |
| KEGG_NON_SMALL_CELL_LUNG_CANCER | 54 | -1.573033879 | 0.018290985 | 0.037003608 | 6953 |
| KEGG_INSULIN_SIGNALING_PATHWAY | 133 | -1.396078623 | 0.018382985 | 0.037003608 | 6335 |
| KEGG_MTOR_SIGNALING_PATHWAY | 50 | -1.543734318 | 0.019464243 | 0.038504583 | 6275 |
| KEGG_CYTOKINE_CYTOKINE_RECEPTOR_INTERACTION | 250 | -1.383005374 | 0.020824807 | 0.04049784 | 2139 |
| KEGG_METABOLISM_OF_XENOBIOTICS_BY_CYTOCHROME_P450 | 55 | 1.450730919 | 0.023556052 | 0.045045785 | 8082 |
| KEGG_SNARE_INTERACTIONS_IN_VESICULAR_TRANSPORT | 37 | -1.562504356 | 0.0262538 | 0.049381609 | 5018 |
| KEGG_PROGESTERONE_MEDIATED_OOCYTE_MATURATION | 83 | -1.456978954 | 0.027358698 | 0.050039192 | 6086 |
| KEGG_ASCORBATE_AND_ALDARATE_METABOLISM | 14 | 1.615130805 | 0.027475648 | 0.050039192 | 722 |
| KEGG_CELL_ADHESION_MOLECULES_CAMS | 125 | -1.404619998 | 0.028248416 | 0.05064272 | 3939 |
| KEGG_VEGF_SIGNALING_PATHWAY | 71 | -1.44245161 | 0.029075382 | 0.051199454 | 5405 |
| KEGG_PATHWAYS_IN_CANCER | 318 | -1.331826326 | 0.029451429 | 0.051199454 | 5041 |
| KEGG_OOCYTE_MEIOSIS | 107 | -1.438064878 | 0.0305583 | 0.052330788 | 4467 |
| KEGG_CALCIUM_SIGNALING_PATHWAY | 172 | 1.275604718 | 0.03193119 | 0.053877704 | 5501 |
| KEGG_CITRATE_CYCLE_TCA_CYCLE | 30 | -1.57216545 | 0.032913055 | 0.054729565 | 5483 |
| KEGG_DRUG_METABOLISM_CYTOCHROME_P450 | 58 | 1.442946039 | 0.034149458 | 0.0559743 | 2343 |
| KEGG_ETHER_LIPID_METABOLISM | 25 | -1.538454523 | 0.037453808 | 0.059653514 | 1829 |
| KEGG_ADHERENS_JUNCTION | 66 | -1.437938732 | 0.037524456 | 0.059653514 | 5415 |
| KEGG_OXIDATIVE_PHOSPHORYLATION | 95 | -1.390580349 | 0.037953864 | 0.059653514 | 7062 |
| KEGG_MAPK_SIGNALING_PATHWAY | 254 | -1.306178008 | 0.040673212 | 0.063063728 | 4763 |
| KEGG_ALZHEIMERS_DISEASE | 138 | -1.356005243 | 0.047765794 | 0.073073284 | 7454 |

# Appendix 6

**Drug prediction**

**Table 6. Drug prediction.**

| search_term | gene | drug | interaction_types | sources |
| --- | --- | --- | --- | --- |
| NPR2 | NPR2 | NESIRITIDE | unknown | TdgClinicalTrial|TEND |
| PDE6D | PDE6D | DIPYRIDAMOLE | inhibitor | ChemblInteractions |
| PDE6D | PDE6D | PENTOXIFYLLINE | inhibitor | ChemblInteractions |
| PDE6H | PDE6H | DIPYRIDAMOLE | inhibitor | ChemblInteractions |
| PDE6H | PDE6H | PENTOXIFYLLINE | inhibitor | ChemblInteractions |
| PDE4B | PDE4B | AMINOPHYLLINE | inhibitor | ChemblInteractions |
| PDE4B | PDE4B | ROFLUMILAST | inhibitor | TdgClinicalTrial|ChemblInteractions |
| PDE4B | PDE4B | PAPAVERINE | inhibitor | TdgClinicalTrial|TEND |
| PDE4B | PDE4B | THEOPHYLLINE | inhibitor | TdgClinicalTrial|ChemblInteractions|TEND |
| PDE4B | PDE4B | OXTRIPHYLLINE | inhibitor | ChemblInteractions |
| PDE4B | PDE4B | FLAVOXATE HYDROCHLORIDE | inhibitor | ChemblInteractions |
| PDE4B | PDE4B | AMLEXANOX | inhibitor | ChemblInteractions |
| PDE4B | PDE4B | INAMRINONE | inhibitor | TdgClinicalTrial|TEND |
| PDE4B | PDE4B | APREMILAST | antagonist|inhibitor | TdgClinicalTrial|ChemblInteractions |
| PDE4B | PDE4B | DIPYRIDAMOLE | inhibitor | ChemblInteractions |
| PDE4B | PDE4B | PENTOXIFYLLINE | inhibitor | TdgClinicalTrial|ChemblInteractions|TEND |
| PDE4B | PDE4B | DYPHYLLINE | inhibitor | TdgClinicalTrial|ChemblInteractions|TEND |
| PDE4B | PDE4B | THEOPHYLLINE SODIUM GLYCINATE | inhibitor | ChemblInteractions |
| PDE4B | PDE4B | ALCOHOL | unknown | PharmGKB |
| PDE4B | PDE4B | CAFFEINE | inhibitor | TdgClinicalTrial|TEND |
| PDE4B | PDE4B | CRISABOROLE | inhibitor | TdgClinicalTrial|ChemblInteractions |

# Appendix 7

**MiRNA and LncRNA**

**Table 7a. Gene-miRNA.**

| Gene | miRNA | miRanda | miRDB | TargetScan | Sum |
| --- | --- | --- | --- | --- | --- |
| ENTPD1 | hsa-miR-545-5p | 1 | 1 | 1 | 3 |
| ENTPD1 | hsa-miR-1183 | 1 | 1 | 1 | 3 |
| PDE4B | hsa-miR-1207-5p | 1 | 1 | 1 | 3 |
| PDE6H | hsa-miR-616-3p | 1 | 1 | 1 | 3 |
| PDE4B | hsa-miR-1200 | 1 | 1 | 1 | 3 |
| ENTPD1 | hsa-miR-508-3p | 1 | 1 | 1 | 3 |
| ENTPD1 | hsa-miR-4273 | 1 | 1 | 1 | 3 |
| PDE4B | hsa-miR-3174 | 1 | 1 | 1 | 3 |
| PDE6D | hsa-miR-873-5p | 1 | 1 | 1 | 3 |
| ENTPD1 | hsa-miR-1-3p | 1 | 1 | 1 | 3 |
| ENTPD1 | hsa-miR-195-5p | 1 | 1 | 1 | 3 |
| POLR2K | hsa-let-7b-3p | 1 | 1 | 1 | 3 |
| PDE6D | hsa-miR-92a-1-5p | 1 | 1 | 1 | 3 |
| PDE6H | hsa-miR-934 | 1 | 1 | 1 | 3 |
| PDE4B | hsa-miR-199a-3p | 1 | 1 | 1 | 3 |
| ENTPD1 | hsa-miR-302b-5p | 1 | 1 | 1 | 3 |
| ENTPD1 | hsa-miR-188-3p | 1 | 1 | 1 | 3 |
| ENTPD1 | hsa-miR-30b-3p | 1 | 1 | 1 | 3 |
| PDE4B | hsa-miR-548u | 1 | 1 | 1 | 3 |
| ENTPD1 | hsa-miR-4282 | 1 | 1 | 1 | 3 |
| ENTPD1 | hsa-miR-766-3p | 1 | 1 | 1 | 3 |
| PDE4B | hsa-miR-940 | 1 | 1 | 1 | 3 |
| PDE4B | hsa-miR-4255 | 1 | 1 | 1 | 3 |
| ENTPD1 | hsa-miR-145-3p | 1 | 1 | 1 | 3 |
| PDE4B | hsa-miR-2113 | 1 | 1 | 1 | 3 |
| ENTPD1 | hsa-miR-4305 | 1 | 1 | 1 | 3 |
| ENTPD1 | hsa-miR-136-5p | 1 | 1 | 1 | 3 |
| PDE4B | hsa-miR-148a-3p | 1 | 1 | 1 | 3 |
| ENTPD1 | hsa-miR-340-5p | 1 | 1 | 1 | 3 |
| ENTPD1 | hsa-miR-583 | 1 | 1 | 1 | 3 |
| ENTPD1 | hsa-miR-107 | 1 | 1 | 1 | 3 |
| PDE6D | hsa-miR-2054 | 1 | 1 | 1 | 3 |
| ENTPD1 | hsa-miR-297 | 1 | 1 | 1 | 3 |
| PDE6H | hsa-miR-539-5p | 1 | 1 | 1 | 3 |
| PDE6H | hsa-miR-3135a | 1 | 1 | 1 | 3 |
| ENTPD1 | hsa-miR-758-3p | 1 | 1 | 1 | 3 |
| ENTPD1 | hsa-miR-223-5p | 1 | 1 | 1 | 3 |
| POLR2K | hsa-let-7f-2-3p | 1 | 1 | 1 | 3 |
| ENTPD1 | hsa-miR-132-3p | 1 | 1 | 1 | 3 |
| ENTPD1 | hsa-miR-1179 | 1 | 1 | 1 | 3 |
| ENTPD1 | hsa-miR-3175 | 1 | 1 | 1 | 3 |
| ENTPD1 | hsa-miR-373-5p | 1 | 1 | 1 | 3 |
| ENTPD1 | hsa-miR-376b-3p | 1 | 1 | 1 | 3 |
| PDE6D | hsa-miR-3148 | 1 | 1 | 1 | 3 |
| ENTPD1 | hsa-miR-490-5p | 1 | 1 | 1 | 3 |
| PDE6H | hsa-miR-600 | 1 | 1 | 1 | 3 |
| PDE4B | hsa-miR-143-5p | 1 | 1 | 1 | 3 |
| PDE6D | hsa-miR-765 | 1 | 1 | 1 | 3 |
| ENTPD1 | hsa-miR-1207-3p | 1 | 1 | 1 | 3 |
| ENTPD1 | hsa-miR-3187-3p | 1 | 1 | 1 | 3 |
| PDE4B | hsa-miR-545-3p | 1 | 1 | 1 | 3 |
| PDE4B | hsa-miR-23b-3p | 1 | 1 | 1 | 3 |
| ENTPD1 | hsa-miR-4292 | 1 | 1 | 1 | 3 |
| ENTPD1 | hsa-miR-548x-3p | 1 | 1 | 1 | 3 |
| PDE6D | hsa-miR-548x-3p | 1 | 1 | 1 | 3 |
| NPR2 | hsa-miR-3126-5p | 1 | 1 | 1 | 3 |
| NPR2 | hsa-miR-3144-5p | 1 | 1 | 1 | 3 |
| PDE4B | hsa-miR-449b-5p | 1 | 1 | 1 | 3 |
| PDE6D | hsa-miR-1183 | 1 | 1 | 1 | 3 |
| PDE4B | hsa-miR-20b-3p | 1 | 1 | 1 | 3 |
| PDE6D | hsa-miR-3173-3p | 1 | 1 | 1 | 3 |
| PDE4B | hsa-miR-21-3p | 1 | 1 | 1 | 3 |
| PDE4B | hsa-miR-345-5p | 1 | 1 | 1 | 3 |
| PDE4B | hsa-miR-222-5p | 1 | 1 | 1 | 3 |
| ENTPD1 | hsa-miR-509-3p | 1 | 1 | 1 | 3 |
| ENTPD1 | hsa-miR-135b-5p | 1 | 1 | 1 | 3 |
| PDE4B | hsa-miR-4272 | 1 | 1 | 1 | 3 |
| PDE4B | hsa-miR-26b-5p | 1 | 1 | 1 | 3 |
| ENTPD1 | hsa-miR-1257 | 1 | 1 | 1 | 3 |
| PDE6H | hsa-miR-92a-2-5p | 1 | 1 | 1 | 3 |
| ENTPD1 | hsa-miR-4291 | 1 | 1 | 1 | 3 |
| ENTPD1 | hsa-miR-599 | 1 | 1 | 1 | 3 |
| ENTPD1 | hsa-miR-4298 | 1 | 1 | 1 | 3 |
| ENTPD1 | hsa-miR-212-3p | 1 | 1 | 1 | 3 |
| ENTPD1 | hsa-miR-1304-5p | 1 | 1 | 1 | 3 |
| PDE4B | hsa-miR-877-3p | 1 | 1 | 1 | 3 |
| PDE4B | hsa-miR-497-3p | 1 | 1 | 1 | 3 |
| POLR2K | hsa-let-7f-1-3p | 1 | 1 | 1 | 3 |
| PDE6D | hsa-miR-331-3p | 1 | 1 | 1 | 3 |
| PDE4B | hsa-miR-3166 | 1 | 1 | 1 | 3 |
| PDE4B | hsa-miR-4277 | 1 | 1 | 1 | 3 |
| PDE6D | hsa-miR-548u | 1 | 1 | 1 | 3 |
| ENTPD1 | hsa-miR-9-5p | 1 | 1 | 1 | 3 |
| ENTPD1 | hsa-miR-2117 | 1 | 1 | 1 | 3 |
| PDE4B | hsa-miR-369-3p | 1 | 1 | 1 | 3 |
| PDE4B | hsa-miR-599 | 1 | 1 | 1 | 3 |
| ENTPD1 | hsa-miR-548a-3p | 1 | 1 | 1 | 3 |
| ENTPD1 | hsa-miR-16-5p | 1 | 1 | 1 | 3 |
| ENTPD1 | hsa-miR-590-3p | 1 | 1 | 1 | 3 |
| ENTPD1 | hsa-miR-630 | 1 | 1 | 1 | 3 |
| POLR2K | hsa-miR-613 | 1 | 1 | 1 | 3 |
| PDE4B | hsa-miR-449a | 1 | 1 | 1 | 3 |
| PDE4B | hsa-miR-1253 | 1 | 1 | 1 | 3 |
| NPR2 | hsa-let-7a-2-3p | 1 | 1 | 1 | 3 |
| PDE6D | hsa-miR-30b-3p | 1 | 1 | 1 | 3 |
| NPR2 | hsa-miR-3145-3p | 1 | 1 | 1 | 3 |
| ENTPD1 | hsa-miR-4297 | 1 | 1 | 1 | 3 |
| ENTPD1 | hsa-miR-576-3p | 1 | 1 | 1 | 3 |
| PDE4B | hsa-miR-524-5p | 1 | 1 | 1 | 3 |
| ENTPD1 | hsa-miR-562 | 1 | 1 | 1 | 3 |
| PDE4B | hsa-miR-663b | 1 | 1 | 1 | 3 |
| ENTPD1 | hsa-miR-518a-5p | 1 | 1 | 1 | 3 |
| PDE4B | hsa-miR-539-5p | 1 | 1 | 1 | 3 |
| PDE4B | hsa-miR-3120-3p | 1 | 1 | 1 | 3 |
| ENTPD1 | hsa-miR-424-5p | 1 | 1 | 1 | 3 |
| PDE4B | hsa-miR-542-5p | 1 | 1 | 1 | 3 |
| ENTPD1 | hsa-miR-943 | 1 | 1 | 1 | 3 |
| POLR2K | hsa-miR-3148 | 1 | 1 | 1 | 3 |
| POLR2K | hsa-miR-2115-3p | 1 | 1 | 1 | 3 |
| ENTPD1 | hsa-miR-519e-5p | 1 | 1 | 1 | 3 |
| PDE6D | hsa-miR-138-2-3p | 1 | 1 | 1 | 3 |
| ENTPD1 | hsa-miR-1294 | 1 | 1 | 1 | 3 |
| ENTPD1 | hsa-miR-149-3p | 1 | 1 | 1 | 3 |
| ENTPD1 | hsa-miR-4276 | 1 | 1 | 1 | 3 |
| ENTPD1 | hsa-miR-4324 | 1 | 1 | 1 | 3 |
| ENTPD1 | hsa-miR-4261 | 1 | 1 | 1 | 3 |
| ENTPD1 | hsa-miR-3116 | 1 | 1 | 1 | 3 |
| POLR2K | hsa-miR-126-5p | 1 | 1 | 1 | 3 |
| PDE4B | hsa-miR-34a-5p | 1 | 1 | 1 | 3 |
| ENTPD1 | hsa-miR-4254 | 1 | 1 | 1 | 3 |
| ENTPD1 | hsa-miR-24-3p | 1 | 1 | 1 | 3 |
| PDE4B | hsa-miR-1284 | 1 | 1 | 1 | 3 |
| ENTPD1 | hsa-miR-542-3p | 1 | 1 | 1 | 3 |
| PDE4B | hsa-miR-361-5p | 1 | 1 | 1 | 3 |
| ENTPD1 | hsa-miR-527 | 1 | 1 | 1 | 3 |
| POLR2K | hsa-miR-206 | 1 | 1 | 1 | 3 |
| ENTPD1 | hsa-miR-1262 | 1 | 1 | 1 | 3 |
| ENTPD1 | hsa-miR-302d-5p | 1 | 1 | 1 | 3 |
| ENTPD1 | hsa-miR-142-3p | 1 | 1 | 1 | 3 |
| PDE6D | hsa-miR-551b-5p | 1 | 1 | 1 | 3 |
| ENTPD1 | hsa-miR-1226-5p | 1 | 1 | 1 | 3 |
| ENTPD1 | hsa-miR-198 | 1 | 1 | 1 | 3 |
| NPR2 | hsa-miR-448 | 1 | 1 | 1 | 3 |
| ENTPD1 | hsa-miR-450b-5p | 1 | 1 | 1 | 3 |
| ENTPD1 | hsa-miR-4316 | 1 | 1 | 1 | 3 |
| ENTPD1 | hsa-miR-206 | 1 | 1 | 1 | 3 |
| PDE4B | hsa-miR-3163 | 1 | 1 | 1 | 3 |
| ENTPD1 | hsa-miR-150-5p | 1 | 1 | 1 | 3 |
| POLR2K | hsa-miR-300 | 1 | 1 | 1 | 3 |
| ENTPD1 | hsa-miR-3179 | 1 | 1 | 1 | 3 |
| ENTPD1 | hsa-miR-515-5p | 1 | 1 | 1 | 3 |
| ENTPD1 | hsa-miR-3136-5p | 1 | 1 | 1 | 3 |
| PDE4B | hsa-miR-338-5p | 1 | 1 | 1 | 3 |
| ENTPD1 | hsa-miR-4330 | 1 | 1 | 1 | 3 |
| ENTPD1 | hsa-miR-29b-2-5p | 1 | 1 | 1 | 3 |
| PDE4B | hsa-miR-1297 | 1 | 1 | 1 | 3 |
| PDE4B | hsa-miR-298 | 1 | 1 | 1 | 3 |
| ENTPD1 | hsa-miR-1256 | 1 | 1 | 1 | 3 |
| ENTPD1 | hsa-miR-607 | 1 | 1 | 1 | 3 |
| PDE4B | hsa-miR-7-5p | 1 | 1 | 1 | 3 |
| NPR2 | hsa-miR-558 | 1 | 1 | 1 | 3 |
| ENTPD1 | hsa-miR-584-5p | 1 | 1 | 1 | 3 |
| PDE6D | hsa-miR-3121-3p | 1 | 1 | 1 | 3 |
| PDE4B | hsa-miR-4276 | 1 | 1 | 1 | 3 |
| ENTPD1 | hsa-miR-892b | 1 | 1 | 1 | 3 |
| PDE4B | hsa-miR-374b-5p | 1 | 1 | 1 | 3 |
| ENTPD1 | hsa-miR-3148 | 1 | 1 | 1 | 3 |
| PDE4B | hsa-miR-199b-3p | 1 | 1 | 1 | 3 |
| PDE6H | hsa-miR-548p | 1 | 1 | 1 | 3 |
| ENTPD1 | hsa-miR-3163 | 1 | 1 | 1 | 3 |
| ENTPD1 | hsa-miR-1224-3p | 1 | 1 | 1 | 3 |
| ENTPD1 | hsa-miR-1200 | 1 | 1 | 1 | 3 |
| POLR2K | hsa-miR-1284 | 1 | 1 | 1 | 3 |
| ENTPD1 | hsa-miR-592 | 1 | 1 | 1 | 3 |
| ENTPD1 | hsa-miR-548c-3p | 1 | 1 | 1 | 3 |
| PDE4B | hsa-miR-26a-5p | 1 | 1 | 1 | 3 |
| PDE4B | hsa-miR-421 | 1 | 1 | 1 | 3 |
| PDE6D | hsa-miR-411-3p | 1 | 1 | 1 | 3 |
| PDE4B | hsa-miR-4328 | 1 | 1 | 1 | 3 |
| ENTPD1 | hsa-miR-125b-5p | 1 | 1 | 1 | 3 |
| PDE4B | hsa-miR-340-5p | 1 | 1 | 1 | 3 |
| PDE6D | hsa-miR-1207-3p | 1 | 1 | 1 | 3 |
| ENTPD1 | hsa-miR-129-5p | 1 | 1 | 1 | 3 |
| ENTPD1 | hsa-miR-140-5p | 1 | 1 | 1 | 3 |
| PDE4B | hsa-miR-641 | 1 | 1 | 1 | 3 |
| ENTPD1 | hsa-miR-568 | 1 | 1 | 1 | 3 |
| ENTPD1 | hsa-miR-558 | 1 | 1 | 1 | 3 |
| ENTPD1 | hsa-miR-4271 | 1 | 1 | 1 | 3 |
| ENTPD1 | hsa-miR-548s | 1 | 1 | 1 | 3 |
| PDE4B | hsa-miR-2115-3p | 1 | 1 | 1 | 3 |
| ENTPD1 | hsa-miR-1827 | 1 | 1 | 1 | 3 |
| ENTPD1 | hsa-miR-3190-3p | 1 | 1 | 1 | 3 |
| NPR2 | hsa-miR-587 | 1 | 1 | 1 | 3 |
| PDE4B | hsa-miR-148b-3p | 1 | 1 | 1 | 3 |
| PDE4B | hsa-miR-145-5p | 1 | 1 | 1 | 3 |
| PDE4B | hsa-miR-23a-3p | 1 | 1 | 1 | 3 |
| ENTPD1 | hsa-miR-485-5p | 1 | 1 | 1 | 3 |
| ENTPD1 | hsa-miR-155-5p | 1 | 1 | 1 | 3 |
| ENTPD1 | hsa-miR-183-3p | 1 | 1 | 1 | 3 |
| ENTPD1 | hsa-miR-3137 | 1 | 1 | 1 | 3 |
| POLR2K | hsa-miR-452-5p | 1 | 1 | 1 | 3 |
| PDE6H | hsa-miR-323a-5p | 1 | 1 | 1 | 3 |
| ENTPD1 | hsa-miR-3149 | 1 | 1 | 1 | 3 |
| ENTPD1 | hsa-miR-4280 | 1 | 1 | 1 | 3 |
| NPR2 | hsa-let-7g-3p | 1 | 1 | 1 | 3 |
| POLR2K | hsa-miR-595 | 1 | 1 | 1 | 3 |
| PDE6D | hsa-miR-592 | 1 | 1 | 1 | 3 |
| ENTPD1 | hsa-miR-3202 | 1 | 1 | 1 | 3 |
| PDE4B | hsa-miR-558 | 1 | 1 | 1 | 3 |
| ENTPD1 | hsa-miR-2054 | 1 | 1 | 1 | 3 |
| ENTPD1 | hsa-miR-4272 | 1 | 1 | 1 | 3 |
| ENTPD1 | hsa-miR-539-5p | 1 | 1 | 1 | 3 |
| ENTPD1 | hsa-miR-586 | 1 | 1 | 1 | 3 |
| PDE4B | hsa-miR-449b-3p | 1 | 1 | 1 | 3 |
| ENTPD1 | hsa-miR-571 | 1 | 1 | 1 | 3 |
| ENTPD1 | hsa-miR-1253 | 1 | 1 | 1 | 3 |
| POLR2K | hsa-let-7a-3p | 1 | 1 | 1 | 3 |
| PDE4B | hsa-miR-3180-5p | 1 | 1 | 1 | 3 |
| ENTPD1 | hsa-miR-1205 | 1 | 1 | 1 | 3 |
| ENTPD1 | hsa-miR-3185 | 1 | 1 | 1 | 3 |
| ENTPD1 | hsa-miR-3119 | 1 | 1 | 1 | 3 |
| ENTPD1 | hsa-miR-3121-3p | 1 | 1 | 1 | 3 |
| ENTPD1 | hsa-miR-205-3p | 1 | 1 | 1 | 3 |
| ENTPD1 | hsa-miR-888-3p | 1 | 1 | 1 | 3 |
| ENTPD1 | hsa-miR-762 | 1 | 1 | 1 | 3 |
| ENTPD1 | hsa-miR-512-3p | 1 | 1 | 1 | 3 |
| POLR2K | hsa-miR-331-5p | 1 | 1 | 1 | 3 |
| ENTPD1 | hsa-miR-508-5p | 1 | 1 | 1 | 3 |
| ENTPD1 | hsa-miR-1302 | 1 | 1 | 1 | 3 |
| POLR2K | hsa-miR-31-3p | 1 | 1 | 1 | 3 |
| ENTPD1 | hsa-miR-135a-5p | 1 | 1 | 1 | 3 |
| ENTPD1 | hsa-miR-497-5p | 1 | 1 | 1 | 3 |
| ENTPD1 | hsa-miR-3130-5p | 1 | 1 | 1 | 3 |
| PDE6D | hsa-miR-379-3p | 1 | 1 | 1 | 3 |
| PDE4B | hsa-miR-34c-5p | 1 | 1 | 1 | 3 |
| PDE4B | hsa-miR-4307 | 1 | 1 | 1 | 3 |
| ENTPD1 | hsa-miR-32-5p | 1 | 1 | 1 | 3 |
| ENTPD1 | hsa-miR-376a-3p | 1 | 1 | 1 | 3 |
| ENTPD1 | hsa-miR-335-5p | 1 | 1 | 1 | 3 |
| PDE4B | hsa-miR-548n | 1 | 1 | 1 | 3 |
| POLR2K | hsa-miR-1-3p | 1 | 1 | 1 | 3 |
| ENTPD1 | hsa-miR-4293 | 1 | 1 | 1 | 3 |
| ENTPD1 | hsa-miR-548l | 1 | 1 | 1 | 3 |
| ENTPD1 | hsa-miR-548k | 1 | 1 | 1 | 3 |
| NPR2 | hsa-miR-634 | 1 | 1 | 1 | 3 |
| NPR2 | hsa-miR-29b-1-5p | 1 | 1 | 1 | 3 |
| POLR2K | hsa-miR-302f | 1 | 1 | 1 | 3 |
| POLR2K | hsa-miR-381-3p | 1 | 1 | 1 | 3 |
| ENTPD1 | hsa-miR-2110 | 1 | 1 | 1 | 3 |
| PDE4B | hsa-miR-506-3p | 1 | 1 | 1 | 3 |
| PDE4B | hsa-miR-142-3p | 1 | 1 | 1 | 3 |
| PDE4B | hsa-miR-1291 | 1 | 1 | 1 | 3 |

**Table 7b. Gene-miRNA.**

| miRNA | lncRNA |
| --- | --- |
| hsa-miR-7-5p | CDR1-AS |
| hsa-miR-1200 | LINC01043 |
| hsa-miR-765 | GAS6-AS1 |
| hsa-miR-143-5p | CTA-414D7.1 |
| hsa-miR-1207-3p | MUC19 |
| hsa-miR-7-5p | RP11-830F9.6 |
| hsa-miR-92a-1-5p | MUC2 |
| hsa-miR-515-5p | TTLL10-AS1 |
| hsa-miR-30b-3p | C10orf91 |
| hsa-miR-149-3p | C10orf91 |
| hsa-miR-323a-5p | RP11-13K12.1 |
| hsa-miR-145-5p | MUC19 |
| hsa-miR-198 | MUC19 |
| hsa-miR-421 | AC079779.7 |
| hsa-miR-223-5p | AC069257.8 |
| hsa-miR-136-5p | CTD-2534I21.9 |
| hsa-miR-143-5p | CTC-265F19.1 |
| hsa-miR-1-3p | LINC01043 |
| hsa-miR-515-5p | AC079779.7 |
| hsa-miR-148a-3p | CITF22-1A6.3 |
| hsa-miR-24-3p | RP11-102K13.5 |
| hsa-miR-545-3p | LINC01070 |
| hsa-miR-7-5p | FLJ35934 |
| hsa-miR-592 | RP11-982M15.8 |
| hsa-miR-766-3p | LINC01070 |
| hsa-miR-765 | RP11-138B4.1 |
| hsa-miR-873-5p | RP5-894D12.5 |
| hsa-let-7a-3p | RP3-323A16.1 |
| hsa-miR-762 | HP09025 |
| hsa-miR-188-3p | CTB-186H2.3 |
| hsa-miR-223-5p | RP3-323A16.1 |
| hsa-miR-1207-3p | RP11-326C3.10 |
| hsa-miR-24-3p | LINC01106 |
| hsa-miR-873-5p | RP11-102K13.5 |
| hsa-miR-198 | RP11-830F9.6 |
| hsa-miR-762 | AP001476.4 |
| hsa-miR-1207-3p | RP11-326C3.14 |
| hsa-let-7a-3p | DPP10-AS2 |
| hsa-miR-1207-3p | TP73-AS1 |
| hsa-miR-2113 | RP11-982M15.8 |
| hsa-miR-765 | RP11-627G23.1 |
| hsa-miR-766-3p | RP13-507P19.2 |
| hsa-miR-515-5p | RP13-507P19.2 |
| hsa-miR-148a-3p | LA16c-306A4.2 |
| hsa-miR-34a-5p | LA16c-306A4.2 |
| hsa-miR-136-5p | RP11-526P6.1 |
| hsa-miR-150-5p | LINC01002 |
| hsa-miR-188-3p | LINC01002 |
| hsa-miR-34a-5p | AP001476.4 |
| hsa-miR-140-5p | MUC2 |
| hsa-miR-7-5p | RP11-338K13.1 |
| hsa-miR-1207-5p | C10orf91 |
| hsa-miR-188-3p | COL4A2-AS2 |
| hsa-miR-1207-5p | RP11-618K13.2 |
| hsa-miR-762 | MUC2 |
| hsa-miR-512-3p | RP11-717I24.1 |
| hsa-miR-1224-3p | RP11-54O7.17 |
| hsa-miR-762 | RP11-394A14.2 |
| hsa-miR-92a-1-5p | RP11-13K12.1 |
| hsa-miR-508-5p | MUC19 |
| hsa-miR-7-5p | RP11-932O9.4 |
| hsa-miR-766-3p | MUC19 |
| hsa-miR-129-5p | RP11-166B2.5 |
| hsa-miR-877-3p | LINC00689 |
| hsa-miR-592 | HPVC1 |
| hsa-miR-149-3p | CTA-941F9.10 |
| hsa-miR-542-5p | RP13-507P19.2 |
| hsa-miR-450b-5p | CTC-265F19.1 |
| hsa-miR-766-3p | CTD-2008P7.3 |
| hsa-miR-30b-3p | MUC2 |
| hsa-miR-1207-5p | LINC00265 |
| hsa-miR-1207-5p | RP11-333E1.2 |
| hsa-miR-766-3p | RP13-580B18.4 |
| hsa-miR-1224-3p | LINC00685 |
| hsa-miR-143-5p | LA16c-306A4.2 |
| hsa-miR-558 | RP11-458F8.4 |
| hsa-miR-150-5p | LINC01165 |
| hsa-miR-198 | RP11-32B5.8 |
| hsa-miR-149-3p | AIRN |
| hsa-let-7a-3p | FAM230B |
| hsa-miR-188-3p | AC084219.4 |
| hsa-miR-766-3p | AC078942.1 |
| hsa-miR-558 | RP11-384K6.6 |
| hsa-miR-1207-5p | AP001476.4 |
| hsa-miR-1207-5p | RP4-539M6.22 |
| hsa-miR-148a-3p | RP11-717I24.1 |
| hsa-miR-515-5p | RP13-580B18.4 |
| hsa-miR-888-3p | FAM95B1 |
| hsa-miR-542-3p | RP4-671O14.7 |
| hsa-miR-421 | LINC01165 |
| hsa-miR-7-5p | AC006019.3 |
| hsa-miR-512-3p | RP1-182D15.2 |
| hsa-miR-542-3p | AC079586.1 |
| hsa-miR-1226-5p | CTB-60B18.18 |
| hsa-miR-943 | AP001476.4 |
| hsa-miR-545-3p | LINC01165 |
| hsa-miR-558 | RP5-991B18.1 |
| hsa-miR-142-3p | MUC2 |
| hsa-miR-1-3p | RP3-470B24.5 |
| hsa-miR-663b | RP11-54O7.17 |
| hsa-miR-149-3p | LINC00265 |
| hsa-miR-1200 | LINC01123 |
| hsa-miR-149-3p | RP11-311F12.1 |
| hsa-miR-558 | LINC00265 |
| hsa-miR-766-3p | RP11-1217F2.15 |
| hsa-miR-888-3p | RP1-27K12.2 |
| hsa-miR-223-5p | LINC00689 |
| hsa-miR-766-3p | CTD-3193O13.12 |
| hsa-miR-542-3p | RP11-157B13.7 |
| hsa-miR-149-3p | LINC00689 |
| hsa-miR-2113 | AC005264.2 |
| hsa-miR-129-5p | AC006548.28 |
| hsa-miR-877-3p | LINC00940 |
| hsa-miR-145-5p | CTD-3099C6.5 |
| hsa-miR-140-5p | MUC19 |
| hsa-miR-873-5p | RP4-539M6.22 |
| hsa-miR-148a-3p | RP1-182D15.2 |
| hsa-miR-515-5p | SPACA6P |
| hsa-miR-223-5p | RP11-426C22.4 |
| hsa-miR-515-5p | AC093642.4 |
| hsa-miR-148a-3p | SNHG14 |
| hsa-miR-223-5p | HPVC1 |
| hsa-miR-126-5p | RP11-164O23.8 |
| hsa-miR-297 | CTA-929C8.6 |
| hsa-miR-542-3p | LINC00917 |
| hsa-miR-1200 | CTD-2008P7.3 |
| hsa-miR-766-3p | RP11-85G18.6 |
| hsa-miR-539-5p | AC018816.3 |
| hsa-miR-1207-5p | RP11-680F20.6 |
| hsa-miR-30b-3p | LINCMD1 |
| hsa-miR-30b-3p | RP11-153F5.7 |
| hsa-miR-766-3p | LINC01022 |
| hsa-miR-1200 | RP11-22M7.2 |
| hsa-miR-515-5p | FAM74A1 |
| hsa-miR-345-5p | RP11-423H2.5 |
| hsa-miR-873-5p | RP11-333E1.2 |
| hsa-miR-145-3p | AC011284.3 |
| hsa-let-7a-2-3p | AC011718.2 |
| hsa-miR-1207-3p | CTD-2006K23.1 |
| hsa-miR-223-5p | RP11-243A14.1 |
| hsa-miR-1207-5p | RP11-867G23.4 |
| hsa-miR-30b-3p | AC011284.3 |
| hsa-miR-766-3p | RP11-142C4.6 |
| hsa-miR-766-3p | LINC01002 |
| hsa-miR-765 | ST20-AS1 |
| hsa-miR-1200 | RP11-627G23.1 |
| hsa-miR-149-3p | CTA-315H11.2 |
| hsa-miR-143-5p | LINC00686 |
| hsa-miR-149-3p | RP11-153F5.7 |
| hsa-miR-149-3p | LINC00173 |
| hsa-miR-758-3p | AC079586.1 |
| hsa-miR-1207-3p | KCNQ1OT1 |
| hsa-miR-590-3p | LINC00240 |
| hsa-miR-150-5p | AC015849.13 |
| hsa-miR-149-3p | TMEM191A |
| hsa-miR-1207-5p | LINC00969 |
| hsa-miR-1207-3p | LINC00588 |
| hsa-miR-143-5p | RP11-154H17.1 |
| hsa-miR-149-3p | RP11-186N15.3 |
| hsa-miR-129-5p | LINC00662 |
| hsa-miR-515-5p | LINC01002 |
| hsa-miR-1207-5p | H19 |
| hsa-miR-1207-5p | RP5-1142A6.2 |
| hsa-miR-7-5p | RP11-394A14.2 |
| hsa-miR-1200 | RP11-1129I3.1 |
| hsa-miR-542-3p | LINC01224 |
| hsa-miR-590-3p | AC005614.3 |
| hsa-miR-539-5p | ZNF883 |
| hsa-miR-766-3p | CTD-2311B13.1 |
| hsa-miR-145-5p | CTA-390C10.9 |
| hsa-miR-198 | CTA-390C10.9 |
| hsa-miR-558 | CITF22-24E5.1 |
| hsa-miR-539-5p | CTC-435M10.10 |
| hsa-miR-331-3p | RP11-44M6.7 |
| hsa-miR-150-5p | RP11-38M8.1 |
| hsa-miR-558 | RP11-503N18.1 |
| hsa-miR-136-5p | SPACA6P |
| hsa-miR-766-3p | CTC-338M12.9 |
| hsa-miR-129-5p | RP11-69I8.2 |
| hsa-miR-30b-3p | TTLL10-AS1 |
| hsa-miR-766-3p | AP001631.9 |
| hsa-miR-1226-5p | RP11-256I23.1 |
| hsa-miR-765 | AC005324.6 |
| hsa-miR-149-3p | CTD-3193O13.1 |
| hsa-miR-1200 | LINC00689 |
| hsa-miR-758-3p | LINC01224 |
| hsa-miR-558 | RP11-638I8.1 |
| hsa-miR-766-3p | TTN-AS1 |
| hsa-miR-873-5p | RP11-96L7.2 |
| hsa-miR-7-5p | LINC00662 |
| hsa-miR-30b-3p | RP11-94C24.13 |
| hsa-miR-590-3p | RP11-762H8.4 |
| hsa-miR-515-5p | CTD-2197I11.1 |
| hsa-miR-762 | C10orf91 |
| hsa-miR-515-5p | RP11-1217F2.15 |
| hsa-miR-21-3p | RP11-130C6.1 |
| hsa-miR-616-3p | RP1-17K7.1 |
| hsa-miR-323a-5p | HCG22 |
| hsa-miR-188-3p | RP11-431K24.1 |
| hsa-miR-485-5p | AP001626.2 |
| hsa-miR-149-3p | MAFG-AS1 |
| hsa-miR-145-5p | RP11-717I24.1 |
| hsa-miR-1207-3p | AC005481.5 |
| hsa-miR-873-5p | RP13-895J2.3 |
| hsa-miR-143-5p | CTD-2015G9.2 |
| hsa-miR-135a-5p | DARS-AS1 |
| hsa-miR-762 | PABPC1L2B-AS1 |
| hsa-miR-129-5p | RP11-67K19.3 |
| hsa-miR-512-3p | RP5-1154L15.1 |
| hsa-miR-765 | ATP2A1-AS1 |
| hsa-miR-1207-5p | LINC01168 |
| hsa-miR-129-5p | REV3L-IT1 |
| hsa-miR-1-3p | GNG12-AS1 |
| hsa-miR-590-3p | AC006548.28 |
| hsa-miR-145-5p | AC015849.16 |
| hsa-miR-30b-3p | RP11-244B22.11 |
| hsa-miR-590-3p | AC093639.1 |
| hsa-miR-1207-5p | AC000095.11 |
| hsa-miR-1226-5p | RP11-655H13.2 |
| hsa-miR-485-5p | LINC00265 |
| hsa-miR-766-3p | CTD-3099C6.5 |
| hsa-miR-515-5p | CTD-3099C6.5 |
| hsa-miR-149-3p | RP11-148K1.12 |
| hsa-miR-335-5p | SLC8A1-AS1 |
| hsa-miR-129-5p | RP5-1125A11.7 |
| hsa-miR-490-5p | CDR1-AS |
| hsa-miR-340-5p | LINC00869 |
| hsa-miR-539-5p | SATB1-AS1 |
| hsa-miR-515-5p | SNHG14 |
| hsa-miR-149-3p | RP11-430G17.3 |
| hsa-miR-129-5p | RP3-508I15.22 |
| hsa-miR-126-5p | RP11-517O13.1 |
| hsa-miR-149-3p | RP11-1348G14.8 |
| hsa-miR-877-3p | RP1-253P7.1 |
| hsa-miR-539-5p | LINC01539 |
| hsa-miR-515-5p | AC084219.4 |
| hsa-miR-943 | CTD-3099C6.5 |
| hsa-miR-515-5p | AC015849.16 |
| hsa-miR-24-3p | LINC01165 |
| hsa-miR-762 | RP5-1171I10.5 |
| hsa-miR-509-3p | CTD-2278I10.1 |
| hsa-miR-129-5p | SEPSECS-AS1 |
| hsa-miR-34a-5p | LVCAT1 |
| hsa-miR-9-5p | RP11-397O4.1 |
| hsa-miR-149-3p | PAX8-AS1 |
| hsa-miR-766-3p | CTD-2008P7.1 |
| hsa-miR-297 | AC084219.4 |
| hsa-let-7a-3p | LPP-AS2 |
| hsa-miR-1224-3p | OR2A1-AS1 |
| hsa-miR-539-5p | RP11-598F7.3 |
| hsa-miR-129-5p | RP11-848P1.3 |
| hsa-miR-590-3p | CTD-2561J22.5 |
| hsa-miR-515-5p | FAM74A7 |
| hsa-miR-129-5p | RP11-486O12.2 |
| hsa-miR-518a-5p | CTD-2521M24.5 |
| hsa-miR-30b-3p | RP11-480I12.10 |
| hsa-miR-1207-3p | RP4-751H13.7 |
| hsa-miR-485-5p | RP11-384K6.6 |
| hsa-miR-129-5p | RP1-283E3.8 |
| hsa-miR-149-3p | RP11-630C16.2 |
| hsa-miR-758-3p | RP11-157B13.7 |
| hsa-miR-766-3p | ABHD11-AS1 |
| hsa-miR-7-5p | CTA-243E7.1 |
| hsa-miR-762 | KRTAP5-AS1 |
| hsa-miR-129-5p | RP11-189E14.3 |
| hsa-miR-485-5p | AC138035.2 |
| hsa-miR-590-3p | LA16c-60D12.2 |
| hsa-miR-758-3p | RP11-15H20.6 |
| hsa-miR-340-5p | RP11-374A4.1 |
| hsa-miR-24-3p | AC078942.1 |
| hsa-miR-539-5p | EGFLAM-AS3 |
| hsa-miR-1207-5p | MIRLET7BHG |
| hsa-miR-149-3p | CTD-2369P2.8 |
| hsa-miR-765 | RP11-570L14.2 |
| hsa-miR-335-5p | RP11-1030E3.1 |
| hsa-miR-129-5p | RP4-794I6.4 |
| hsa-miR-590-3p | RP11-638L3.1 |
| hsa-miR-940 | RP11-458F8.4 |
| hsa-miR-1205 | RP3-470B24.5 |
| hsa-miR-766-3p | RP11-347H15.4 |
| hsa-let-7f-2-3p | FAM230B |
| hsa-miR-92a-2-5p | CTD-2619J13.14 |
| hsa-miR-940 | AP001476.4 |
| hsa-miR-940 | LINC00265 |
